# Genetic Deletion of Mst1 Alters T Cell Function and Protects against Autoimmunity

**DOI:** 10.1371/journal.pone.0098151

**Published:** 2014-05-22

**Authors:** Konstantin V. Salojin, Brian D. Hamman, Wei Chun Chang, Kanchan G. Jhaver, Amin Al-Shami, Jeannette Crisostomo, Carrie Wilkins, Ann Marie Digeorge-Foushee, Jason Allen, Nita Patel, Suma Gopinathan, Julia Zhou, Amr Nouraldeen, Theodore C. Jessop, Jeffrey T. Bagdanoff, David J. Augeri, Robert Read, Peter Vogel, Jonathan Swaffield, Alan Wilson, Kenneth A. Platt, Kenneth G. Carson, Alan Main, Brian P. Zambrowicz, Tamas Oravecz

**Affiliations:** Lexicon Pharmaceuticals, Inc, The Woodlands, Texas, United States of America; Uniform Services University of the Health Sciences, United States of America

## Abstract

Mammalian sterile 20-like kinase 1 (Mst1) is a MAPK kinase kinase kinase which is involved in a wide range of cellular responses, including apoptosis, lymphocyte adhesion and trafficking. The contribution of Mst1 to Ag-specific immune responses and autoimmunity has not been well defined. In this study, we provide evidence for the essential role of Mst1 in T cell differentiation and autoimmunity, using both genetic and pharmacologic approaches. Absence of Mst1 in mice reduced T cell proliferation and IL-2 production in vitro, blocked cell cycle progression, and elevated activation-induced cell death in Th1 cells. Mst1 deficiency led to a CD4^+^ T cell development path that was biased toward Th2 and immunoregulatory cytokine production with suppressed Th1 responses. In addition, Mst1^−/−^ B cells showed decreased stimulation to B cell mitogens in vitro and deficient Ag-specific Ig production in vivo. Consistent with altered lymphocyte function, deletion of Mst1 reduced the severity of experimental autoimmune encephalomyelitis (EAE) and protected against collagen-induced arthritis development. Mst1^−/−^ CD4^+^ T cells displayed an intrinsic defect in their ability to respond to encephalitogenic antigens and deletion of Mst1 in the CD4^+^ T cell compartment was sufficient to alleviate CNS inflammation during EAE. These findings have prompted the discovery of novel compounds that are potent inhibitors of Mst1 and exhibit desirable pharmacokinetic properties. In conclusion, this report implicates Mst1 as a critical regulator of adaptive immune responses, Th1/Th2-dependent cytokine production, and as a potential therapeutic target for immune disorders.

## Introduction

Mst1, also known as serine/threonine kinase 4 and kinase responsive to stress 2, is a MAPK kinase kinase kinase (MAP4K) and a member of the germinal center kinase subfamily of sterile20-like kinases. Mst1 has been implicated in regulating cell cycle and apoptosis in various species [1], [2], [3], [4]. Hippo (Hpo) kinase, the Drosophila ortholog of human Mst1/2, controls growth and development by phosphorylating the protein kinase Warts [1], [5]. In mammals, Mst1 regulates a number of signaling events upstream of JNK, p38, histone 2B (H2B), large tumor suppressor homolog/Sav/Mps one binder KL1 (MOBKL1), FoxO1 and 3, and AKT (reviewed in [6]). Mst1 is both a target and an activator of the caspase cascade that induces apoptosis [4], [6], [7], [8], [9]. In vitro overexpression of Mst1 and its dimerization activates the MAPK kinase 4 (MKK4)/JNK signaling pathway and caspase-3 and -9, leading to apoptosis [4], [7], [8]. Moreover, phosphorylation of H2B at serine 14 by Mst1 is associated with apoptotic chromatin condensation [10], [11].

Evidence has emerged in recent years that Mst1 is a component of intricate signaling pathways controlling lymphocyte function. Both human and mouse Mst1 genes are preferentially expressed in T and B lymphocytes [12], [13]. Several reports have implicated Mst1 in various aspects of lymphocyte function-associated Ag-1 (LFA-1)-mediated cell polarity, adhesion and trafficking, including homing of lymphocytes to target organs and thymocyte emigration [14], [15], [16], [17]. Mst1 deficient mice display an accumulation of mature lymphocytes in the thymus and low numbers of naive T cells in the peripheral lymphoid organs due to dysregulated chemotaxis and apoptosis [13], [14], [16], [17], [18]. In addition to its effect on cell trafficking and survival, Mst1 may also control Ag receptor-induced activation of naïve T cells by phosphorylating the cell cycle inhibitory proteins MOBKL1A and B [13]. In the context of naive T cell proliferation, the Mst1/RASSF5 (RAS association domain family protein 5)/RAPL (regulator for cell adhesion and polarization enriched in lymphoid tissue) signaling complex was described as a negative regulator of T cell function [13]. However, further studies of Mst1 deficient T cells demonstrated that Mst1 may be regulating lymphocyte survival by protecting T lymphocytes from cellular oxidative stress and controlling the expression of the IL7 receptor [13], [18]. Mst1 was shown to regulate T cell survival and naive T cell homeostasis in the periphery by activating the FoxO1 and 3 transcriptional factors and their downstream targets, Sod2 and catalase, involved in the regulation of cellular oxidative stress [18].

These findings prompted us to examine the role of Mst1 in T cell-mediated adaptive immune responses, Th1/Th2 development, and autoimmunity, using genetic and pharmacologic approaches. We report that Mst1 controls multiple aspects of lymphocyte physiology, including cell cycle progression and proliferation, Th1/Th2-dependent cytokine production, and apoptosis. Mst1 plays a nonredundant role in autoimmunity and is critical for disease induction in a number of autoimmune and inflammatory disease models.

## Materials and Methods

### Ethics statement

Procedures involving animals were conducted in conformity with the Institutional Animal Care and Use Committee guidelines that are in compliance with the state and federal laws and the standards outlined in the Guide for the Care and Use of Laboratory Animals. The Animal Care and Use Committee at Lexicon Pharmaceuticals, Inc., reviewed and approved all experimental procedures involving mice (Permit Numbers: 071, 076, 116, and 188). All mice analyzed were maintained in an AAALAC-accredited animal facility at Lexicon Pharmaceuticals, Inc. (accreditation unit #001025).

### Generation of Mst1 mutant mice

The Mst1 targeting vector was derived using long-range PCR to generate the 5′ and 3′ arms of homology using 129S5 ES cell DNA as a template. The 6071bp 5′ arm was generated using primers Mst1-19 [5′- AATCGTCGACACCTGCCTATAGAGCCGAGTT-3′] and Mst1-20 [5′- AATTGGCGCGCCGCCATAAGACCTGCGTTTGGG-3′] and cloned using the TOPO (Invitrogen) cloning kit. The 3194 bp 3′ arm was generated using primers Mst1-23 [5′- AATTGGCGCGCCCCTTGGGTCTCTGCTCTATACTCA-3′] and Mst1-24 [5′- AATCTCGAGGGAAACTGGTTGGCTTAATTG-3′] and cloned using the TOPO cloning kit. The 5′ arm was excised from the holding plasmid using Sal I and Asc I. The 3′ arm was excised from the holding plasmid using Asc I and Xho I. The arms were ligated to an Asc I prepared selection cassette containing a β-galactosidase-neomycin marker and inserted into a Sal I/Xho I cut pKO Scrambler vector (Stratagene) to complete the Mst1 targeting vector which results in the deletion of coding exons 3 and 4. The Not I linearized targeting vector was electroporated into 129S5 ES cells (Lex2). G418/FIAU resistant ES cell clones were isolated, and correctly targeted clones were identified and confirmed by Southern analysis using a 369 bp 5′ external probe (36/34), generated by PCR using primers Mst1-36 [5′-GACTCAGTGGCTAATGATGG-3′] and Mst1-34 [5′-TCTGAGATGCTAACTAGTAGG-3′] and a 478 bp 3′ internal probe (17/39), amplified by PCR using primers Mst1-17 [5′-TTCTGATGTGGATCTCAGCC-3′] and Mst1-39 [5′-GGAGTAAGCTGTGCACACAGG-3′]. Southern analysis using probe 36/34 detected a 17.2 Kb wild type band and 14.4 Kb mutant band in Avr II digested genomic DNA while probe 17/39 detected a 4.7 Kb wild type band and 6.4 Kb mutants in BamH I digested genomic DNA.

Details of the phenotypic analysis applied to all knockout lines generated in our facilities were described in ref. [19]. It includes clinical diagnostic and pathology tests that query therapeutically relevant aspects of mammalian physiology. Experiments were performed on mice of mixed genetic background (C57Bl/6-Albino/129SvEv) representing both sexes of littermate mutant and WT animals, or C57BL/6J mice.

### Reagents

Reagents were obtained as follows: poly(I:C) from InvivoGen (San Diego, CA); anti-CD40 (HM40-3 clone) from BD Pharmingen (San Diego, CA); AffiniPure F(ab')2 fragment goat anti-mouse IgM (µ chain specific) from Jackson ImmunoResearch Laboratories (West Grove, PA). Con A, LPS from Salmonella Minnesota, ionomycin calcium salt, PMA were purchased from Sigma-Aldrich (St. Louis, MO).

### Peripheral blood analysis

Peripheral blood samples were collected from the retroorbital plexus of mice anesthetized with isoflurane. Flow cytometry analysis of lymphocytes subsets in whole blood was performed as previously described [20], using a Becton Dickinson FACSCalibur flow cytometer with CellQuest software (Becton Dickinson San Jose, CA).

### Splenic T and B cell purification and generation of Th1/Th2 polarized cells in vitro

T and B cells were purified from spleens of 6- to 20-week old WT or Mst1^−/−^ mice by immunomagnetic negative selection (EasySep mouse T and B cell negative selection enrichment kits) to greater than 95% purity, according to the manufacturer's protocol (STEMCELL Technologies, San Diego, CA). The cells were plated in triplicates in 96-well round-bottom plates at a concentration of 1×10^5^ cells per well in RPMI 1640 medium supplemented with 10% FBS, 10 mM HEPES buffer, 2 mM L-glutamine, 100 U/ml penicillin, 0.1 mg/ml streptomycin, and 50 µM 2-ME (c-RPMI; all Gibco Life Technologies, Grand Island, NY).

Th1 and Th2 polarized in vitro cultures were generated from naive (CD62L^high^CD44^low^) CD4^+^ splenic T cells isolated as above by culturing at 37°C and 5% CO_2_ in 6-well plates pre-coated with 2 µg/ml CD3ε mAb (145-2C11 clone) in the presence of soluble 2 µg/ml CD28 mAb (37.51 clone) and 25 U/ml mouse IL-2 for 6 days. For generation of Th1 cells, 10 ng/ml mouse IL-12 and 10 µg/ml anti-mouse IL-4 (clone 11B11; BD Pharmingen) were added to the cultures. For Th2 cells, 20 ng/ml mouse IL-4, 10 µg/ml anti-mouse IL-12, and 10 µg/ml anti-mouse IFN-γ (clone R46A2) were added to the cultures.

### T and B cell proliferation and stimulation assays

T and B cell-specific stimuli were added to the plates at various concentrations as shown in the figure legends. Following incubation for 48 h at 37°C, 5% CO_2_, 40 µl of cell supernatants were harvested from each well, and the plates were pulsed with 1 µCi/well [^3^H]-thymidine (GE Healthcare, Piscataway, NJ) for 16–18 hrs to assess cell proliferation by [^3^H]-thymidine uptake. The incorporated radioactivity was determined by liquid scintillation counting using an automated beta counter. The concentration of cytokines in the supernatants was determined using cytometric bead array (CBA)-based mouse cytokine Flex Sets (BD Biosciences, Mountain View, CA), according to the manufacturer's instructions.

### Allogeneic mixed lymphocyte reaction (MLR)

Irradiated T cell-depleted BALB/c (H-2d) splenocytes were used as stimulator cells. The cells were resuspended in c-RPMI and irradiated with 3000 cGy of γ-radiation from a cesium ^137^Cs source to block their response to the responder cells. C57Bl/6-Albino/129SvEv (H-2b) responder T cells were isolated as described above. A total of 1×10^5^ responder cells were combined with the indicated numbers of irradiated BALB/c stimulator cells in 96-well microtiter flat bottom plates (Fisher Scientific, (Pittsburgh, PA), in triplicate, and incubated at 37°C and 5% CO_2_ for 96 hrs. The MLRs were quantitated by pulsing the plates with 1 µCi/well [^3^H]-thymidine (GE Healthcare) for the last 6 hrs of the assay, and counting the incorporation of [^3^H]-thymidine in a beta-counter.

### FACS analysis of lymphocyte activation, apoptosis, and cytokine production by T cells

Splenic T cells (1.3×10^6^/2 ml/well) were stimulated in 24-well plates with plate-bound 1-5 µg/ml CD3ε mAb in the presence or absence of 1 µg/ml soluble CD28 mAb. The cells were collected and stained for lymphocyte activation and apoptosis markers (CD25, CD44, CD69, CD62L, CD95, CD127, CD178, CD279) according to the FACS staining protocol described above. The expression of the markers was examined by flow cytometry.

For intracellular cytokine staining, lymphocytes were restimulated for 4–5 hrs at 37°C with Leukocyte Activation Cocktail (BD Biosciences), containing PMA, ionomycin, and BD GolgiStop protein transport inhibitor. Activated cells were harvested, fixed and stained with Abs to mouse CD4 (clone RM4-5), TNF (clone MP6-XT22), IFN-γ (clone XMG1.2), IL-2 (clone JES6-5H4), IL-4 (clone 11B11), IL-10 (clone JES5-16E3), IL-13 (clone eBio13A), IL-17A (clone TC11-18H10.1), Tbet (clone eBio4B10), GATA3 (clone L50-823), and bcl-2 (clone 3F11), using an intracellular cytokine staining kit from BD Biosciences. Data was acquired and analyzed using a Becton Dickinson FACSCalibur flow cytometer with CellQuest software.

To measure activation-induced cell death (AICD) in vitro, T cells were stimulated as above, stained with APC Annexin V and 7-AAD (both from BD Biosciences) for 15 min at RT (25°C) in the dark, according to the manufacturer's protocol, and analyzed by flow cytometry within 1 hr. Identification of late apoptotic/necrotic cells and cells undergoing apoptosis was accomplished by gating on high- and low-level 7-AAD-positive cells in gated populations of Annexin V^+^ T cells.

### Cell cycle compartment analysis in vitro

Cell cycle progression and DNA synthesis in T cells were determined by analyzing the total DNA content and incorporated BrdU levels using the BrdU Flow kit (BD Biosciences), according to the manufacturer's protocol. Briefly, splenic T cells (1.3×10^6^/2 ml/well) were stimulated for 36–48 hrs in 24-well plates with plate-bound 1–5 µg/ml CD3ε mAb in the presence or absence of 1 µg/ml soluble CD28 mAb. BrdU was added at a final concentration of 10 µM for the final 4 hours of stimulation. The cells were harvested, stained with anti-BrdU APC and 7-AAD, and analyzed by flow cytometry.

The G1, S, and G2-M compartments were also examined by flow cytometry after staining with propidium iodide (PI). Briefly, isolated T cells were stimulated as above, collected and stored at -20°C in 70% (v/v) ethanol for at least 12–18 hrs prior to staining. The cells were washed in 5 ml PBS twice, stained for 30 min at RT in the dark in 0.5 ml of PI/RNase Staining Buffer (BD Pharmingen), and analyzed in the PI/RNase solution by flow cytometry.

### Anti-OVA Ig production in vivo

Mice were immunized intraperitoneally with 100 µl of an emulsion of equal volumes of chicken OVA (Fraction V; 2 mg/ml in saline; Sigma-Aldrich) mixed 1∶1 with CFA containing 1 mg/ml *Mycobacterium tuberculosis* (Sigma). Serum was collected prior to OVA challenge and 14 days after immunization. Serum levels of OVA-specific IgG1 and IgG2a were measured by OVA-specific ELISA (Cayman Chemical Company, Ann Arbor, MI), according to the manufacturer's protocol. The standard curves for each isotype were created by measuring the absorbance of different concentrations of purified mouse Ig standards.

### Induction and evaluation of experimental autoimmune encephalomyelitis (EAE)

EAE was induced with an adaptation of the method of Bettelli et al. [21]. Eight to 16 weeks old Mst1^−/−^ and Mst1^+/+^ littermates were immunized subcutaneously with a total of 300 µg MOGp35–55 emulsified in CFA containing 250 µg heat-inactivated *Mycobacterium tuberculosis* H37Ra (BD Biosciences). Immediately following the injection of the emulsified Ag (day 0), each mouse received one intravenous injection of 500 ng of *Pertussis* toxin (List Biological Laboratories, Campbell, CA). Disease severity was scored on a scale of 0 to 8 as previously described [22].

For histopathologic analysis, mouse tissues were fixed in neutral buffered 4% formalin, spinal column segments were decalcified with Cal-Rite (Thermo Scientific, Waltham, MA), embedded in paraffin, sectioned at 4 to 6 µm, and stained with H&E. The anatomic regions of brain, brain stem, cervical cord, thoracic cord and lumbar cord (3–5 sections per region) were scored for inflammatory and degenerative lesions according to the following scale: 0 =  absent, 1 = minimal, 2  =  conspicuous focal, 3  =  prominent focal, 4  =  marked multifocal, 5  =  severe diffuse. Sections from animals in different groups were scored in staggered order to prevent score bias.

Mononuclear infiltrating cells were isolated from the spinal cord of WT and Mst1^−/−^ mice with EAE on day 15 after immunization, as previously described [23], [24]. Mice were anesthetized and perfused intracardially with ice-cold 1X HBSS. Mononuclear cells were separated over discontinuous 70%/30% percoll gradients from spinal cord cell suspensions after incubation with 1 mg/ml collagenase II (Sigma-Aldrich) at 37°C for 20 min, and subjected to flow cytometry.

### CD4^+^ T cell transfer into Rag2^−/−^ mice

CD4^+^ T cells were isolated from the spleens of WT or Mst1^−/−^ mice using the EasySep mouse CD4^+^ T negative selection enrichment kit (STEMCELL Technologies). The purity of the isolated CD4^+^ T cells was more than 95%, as determined by flow cytometry. CD4^+^ T cells (15×10^6^ per mouse) were injected into the tail vein of nonirradiated Rag2^−/−^ mice (Taconic Farms, Germantown, NY) as described previously [25]. To induce EAE, the reconstituted Rag2^−/−^ mice were immunized subcutaneously with a total of 300 µg MOGp35–55 emulsified in CFA containing 500 µg heat-inactivated *Mycobacterium tuberculosis* H37Ra. On the day of immunization and 48 hours later, each mouse received one intravenous injection of 500 ng of *Pertussis* toxin. The immunization was repeated on day 8 after CD4^+^ T cell transfer.

### T cell-dependent MOG-specific recall responses in MOGp35–55-immunized Mst1^−/−^ mice

Single-cell suspensions of splenocytes and lymph node cells were plated in 96-well round-bottom plates at a concentration of 1×10^5^ cells per well in c-RPMI, stimulated with a range of concentrations of MOGp35–55 for 72 hrs, and assayed for proliferation by measuring mean incorporation of thymidine in DNA in triplicate wells pulsed with 1 µCi/well [^3^H]-thymidine (GE Healthcare) for the last 16 hrs of the assay. The concentration of cytokines in supernatants was determined at 48 hrs of culture using CBA-based mouse cytokine Flex Sets (BD Biosciences), according to the manufacturer's instructions.

### Quantitative RT-PCR (qPCR)

Total RNA was prepared from spinal cord homogenates using the RNeasy mini-kit protocol (Qiagen, Hilden, Germany), snap frozen in liquid nitrogen and stored at −80°C. The TaqMan reverse transcription reagents (High Capacity cDNA Archive kit; Applied Biosystems, Foster City, CA) were used to synthesize first-strand cDNA from total RNA samples. cDNA amplification was performed on ABI 7500 cycler using TaqMan Mix real-time PCR mix (all from Applied Biosystems) and mouse sequence-specific primers (gene name, accession #, and Applied Biosystems probe ID corresponding to each primer were as follows: tnf, NM_013693, Mm99999068_m1; ifng, NM_008337.3, Mm01168134_m1; IL2, NM_008366.2, Mm00434256_m1; IL4, NM_021283.2, Mm00445260_m1; IL10, NM_010548.2, Mm00439614_m1; Gapdh, NM_008084.2, Mm99999915_g1). Gene expression was calculated using GAPDH as an internal control to normalize for differences in the amount of total RNA in each sample.

### Collagen-induced arthritis (CIA)

Eight to 16 week old Mst1^−/−^ and WT control mice were immunized intradermally at several sites into the base of the tail with 100 µg of chicken type II collagen (CII; Sigma-Aldrich) in CFA containing 2.5 mg/ml *M. tuberculosis* (BD Biosciences), followed by a repeat booster intradermal injection of CII (100 µg emulsified in CFA) given 3 weeks after the primary immunization. Mice were monitored daily for signs of arthritis, and disease severity scores were assessed by a visual scoring of 0 to 4 as described previously [26]. Total disease severity scores were recorded as a sum of visual scores for four limbs. In addition to visual scoring, paw thickness was measured with a micrometer caliper. Thickness values for the two forelimbs were averaged, and the extent of swelling was calculated by subtracting the baseline values of the first measurement from the values of subsequent measurements.

To visualize bone abnormalities in the paw joints of mice with arthritis, we used three-dimensional X-ray microcomputed tomography (µCT) (Scanco Medical µCT40, with 55 keV X-ray energy, 20 µm voxel dimensions and 3D image analysis software).

For histopathologic analysis, mouse tissues were fixed in neutral buffered 4% formalin, decalcified with Cal-Rite, embedded in paraffin, sectioned at 4 to 6 µm collecting 3 step sections spaced at 50–200 µm, and stained with H&E. Joints were scored for lesions of arthritis and synovitis with a single overall score assigned to each section according to the following scale: 0  =  lesions absent to minimal and focal; 1  =  mild focal to diffuse synovial hypertrophy and/or hyperplasia; 2  =  prominent distribution mild to moderate synovial hyperplasia with less stromal involvement; 3  =  prominent regional to widespread synovial changes with strong stromal proliferation; 4  =  severe with joint and/or bone destruction (erosion/ulceration articular cartilage, suppuration and periosteal new bone). Sections from animals in different groups were scored in staggered order to prevent score bias.

### Statistical analysis

The normality of results was evaluated by the Kolmogorov–Smirnov test. Unless otherwise stated, statistical significance of group differences between WT and Mst1^−/−^ mice was analyzed using the two-sample Student's *t* test. Statistical differences in mean daily clinical scores and histology scores in mice with EAE or CIA were analyzed by the nonparametric Mann–Whitney U test or Kruskal–Wallis test with Dunnett's multiple-comparison *post hoc* analysis. The EAE onset times in WT and Mst1^−/−^ mice were compared using a Kaplan-Meier analysis followed by the log rank test. Differences were considered statistically significant at *P* values <0.05.

## Results

### Mst1 deficiency leads to altered distribution of leukocyte subsets and reduced in vitro T cell activation

**Figure 1:**
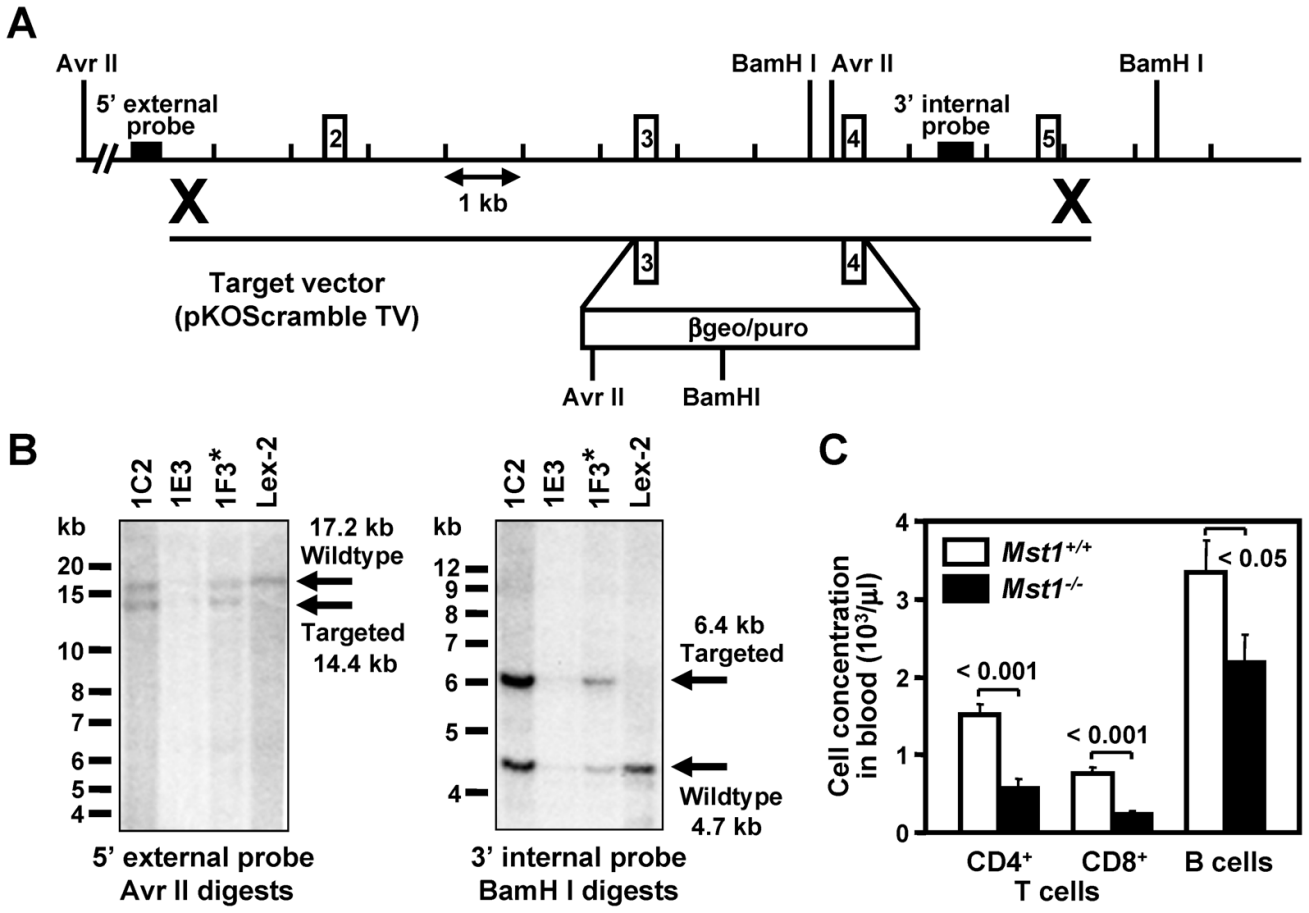
Targeted disruption of the Mst1 gene locus. (**A**) Targeting strategy used to disrupt the Mst1 locus. Homologous recombination (represented by ×) between the targeting vector and the Mst1 gene results in the replacement of exons 3–4 with the selection cassette. (**B**) Southern hybridization indicating proper gene targeting in the embryonic stem cell clones. Clone 1F3 was selected for blastocyst injections to generate chimeric animals which were bred to C57BL/6 (albino) females, and the resulting heterozygous offspring were interbred to produce homozygous Mst1 deficient mice; Lex-2 represents untransfected embryonic stem cell DNA. (**C**) The effect of Mst1 disruption on peripheral lymphocyte subsets. Peripheral blood cell subsets were stained with a panel of lymphocyte lineage-specific mAbs and quantitated by FACS. Values are expressed as mean ± SEM; n = 9 per group. Student's *t* test was used for group comparison.

We generated Mst1 deficient mice by targeted disruption of the murine Mst1 gene (GenBank accession no. NM_021420) by homologous recombination in ESCs [26], [27], followed by germ line transmission of the mutant gene into chimeric, heterozygous (Mst1^+/−^), and homozygous mice (Fig. 1A). Southern hybridization analysis demonstrated the targeted mutation in embryonic stem cells (ESC) (Fig. 1B). Western blot analysis confirmed the absence of Mst1 and showed unaltered levels of Mst2 in splenocyte and kidney cell protein extracts from Mst1^−/−^ mice (Fig. S1). Mst1 deficiency did not affect mouse development and we did not observe any obvious differences between Mst1^−/−^ mice and WT controls in a battery of observational and diagnostic tests designed to detect developmental and endocrinological abnormalities.

In agreement with previously published reports [13], [14], [17], [18], we observed lower CD4^+^ and CD8^+^ T cell counts in the peripheral blood of Mst1^−/−^ mice when compared to those of WT littermates (Fig. 1C). Mst1^−/−^ mice also exhibited a marked decrease in B lymphocyte counts. B and T (CD4^+^ and CD8^+^) lymphocyte counts were also significantly lower in the peripheral lymphoid organs (spleen and lymph nodes) of Mst1^−/−^ mice (data not shown). These results demonstrated that Mst1 plays an important role in the maintenance of normal levels of T and B cell subsets in the periphery.

**Figure 2:**
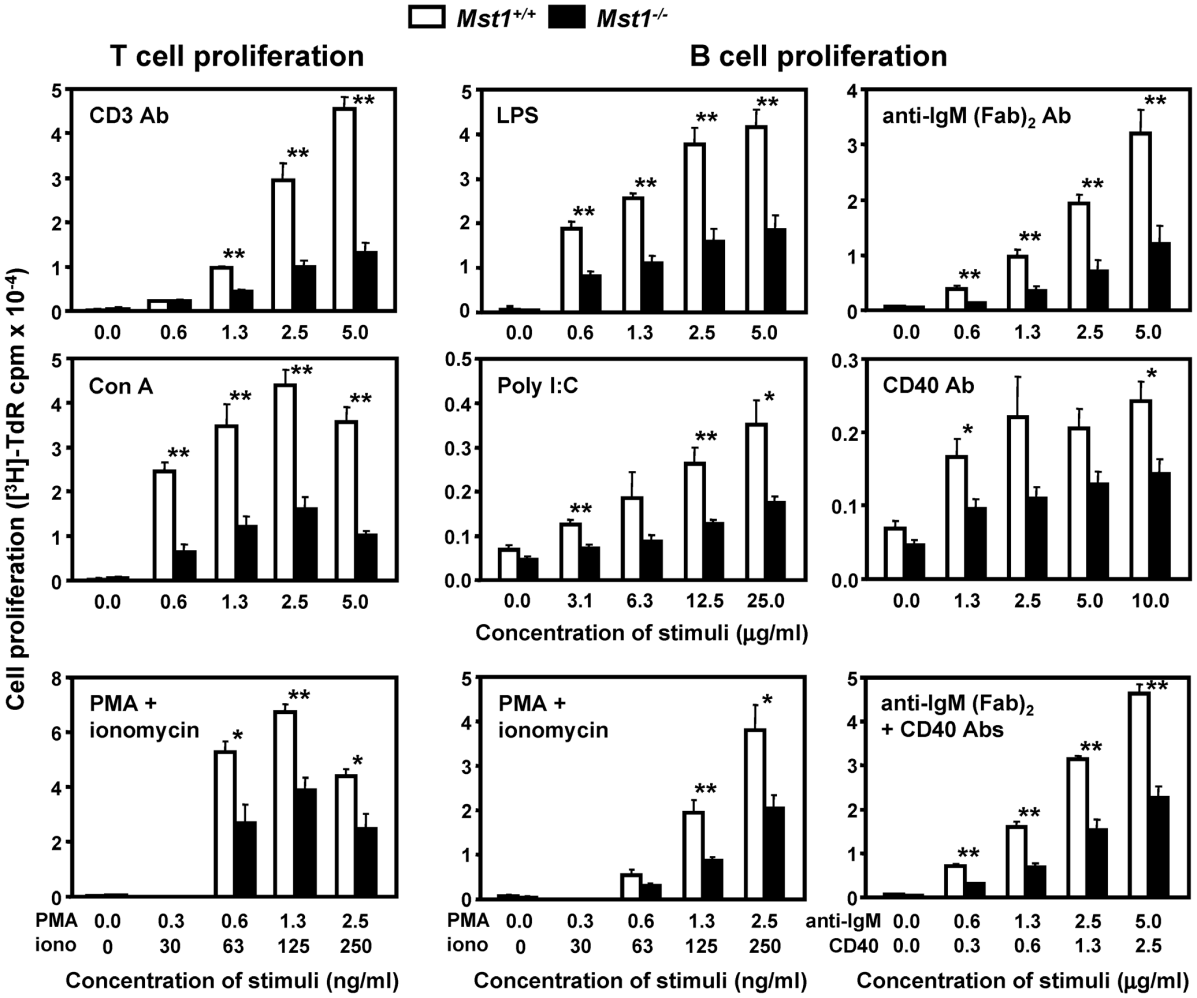
Deficient proliferation of Mst1^−/−^ T and B cells in vitro. Single cell suspensions of splenic T and B lymphocytes were stimulated with various T and B cell-specific stimuli at the indicated concentrations. Cell proliferation was assessed by [^3^H]-thymidine incorporation after 64–66 hrs of stimulation. Data are expressed as the mean ± SEM of counts per minute (cpm) in triplicate cultures (n = 5 per genotype). Values are expressed as mean ± SEM. * (p<0.05) and ** (p<0.01) indicate significant differences in comparison to WT animals (*t* test).

**Figure 3:**
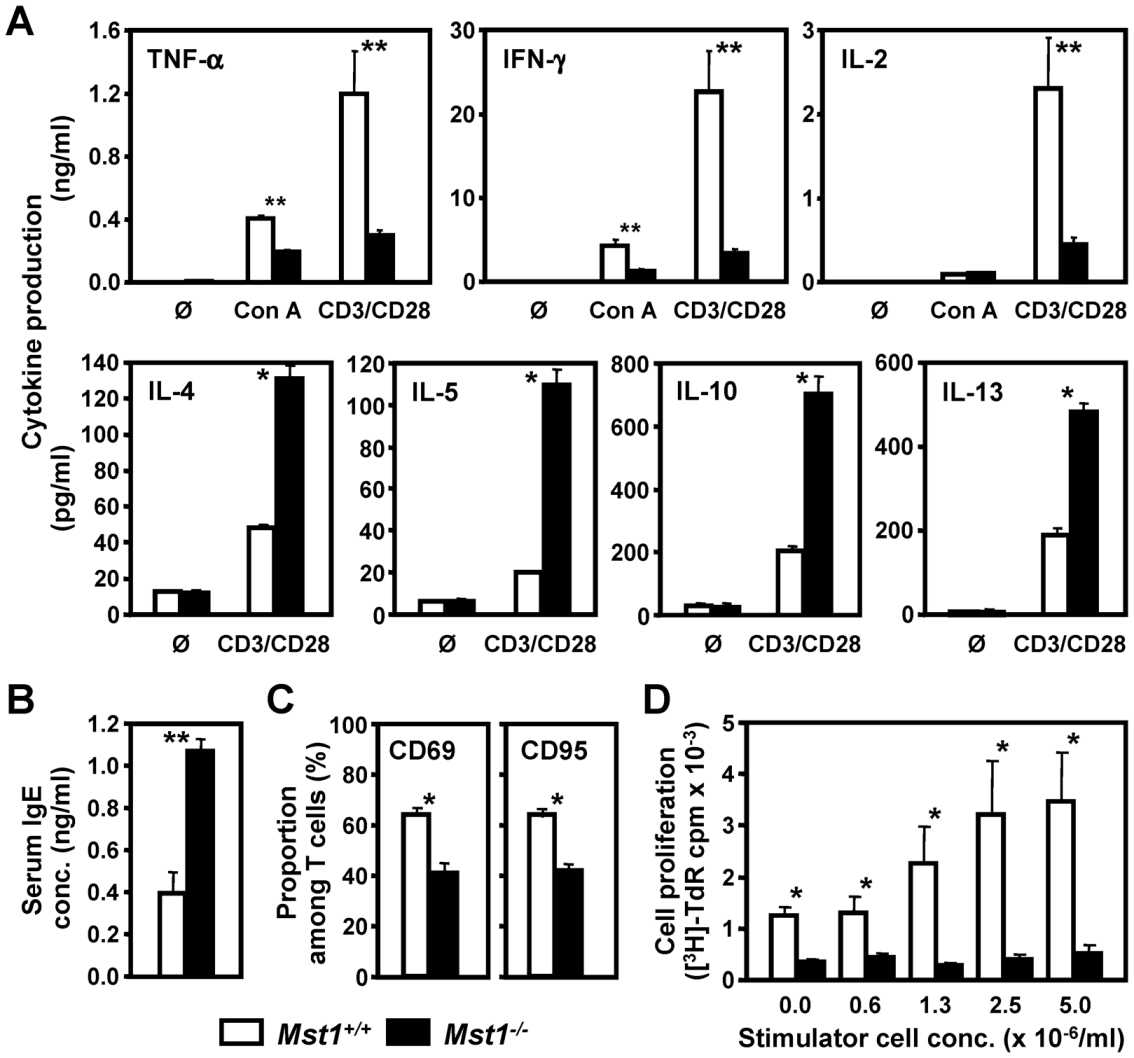
Altered immune responses of activated Mst1^−/−^ T cells in vitro. (**A**) Th1 and Th2 cytokine production by splenic T cells from WT and Mst1^−/−^ mice was examined 48 hrs after stimulation with Con A (2.5 µg/ml) or mAbs to CD3 and CD28 (both at 1 µg/ml). (**B**) IgE concentration was measured by ELISA in serum samples of WT (n = 8) and Mst1^−/−^ mice (n = 10). (**C**) Deficient upregulation of lymphocyte activation markers on Mst1^−/−^ T cells. Splenic T cells were stimulated with plate-bound CD3 mAb (1 µg/ml) for 48 hrs, stained for CD69 and CD95 (Fas/APO-1), and analyzed by flow cytometry (n = 5 per genotype). (**D**) Suppression of Mst1^−/−^ T cell proliferative responses in allogeneic MLR in vitro. Splenic C57Bl/6-Albino/129SvEv (H-2b) Mst1^−/−^ and WT T cells (responder cells; 5 mice per genotype) were stimulated with the indicated numbers of MHC-mismatched irradiated stimulator cells obtained from Balb/c mice (H-2d). Proliferation of the responder cells was assessed by [^3^H]-thymidine incorporation after 90 hrs of culture. Values and statistical significance are expressed as in Fig. 2 and represent results of at least two independent experiments.

To investigate the role of Mst1 in lymphocyte activation in vitro, we measured cell proliferation and cytokine production of WT and Mst1^−/−^ T and B cell cultures stimulated with various T and B cell-specific polyclonal activators and nonspecific mitogens. Proliferation of T cells was markedly reduced in the absence of Mst1 after activation with CD3 mAb, Con A, and a mixture of PMA and ionomycin (Fig. 2). Furthermore, Mst1^−/−^ B cells showed decreased responses to B cell stimuli in vitro (Fig. 2). Deletion of Mst1 also significantly inhibited the release of IL-2, IFN-γ, and TNF-α in Mst1^−/−^ T cell cultures after stimulation via the CD3/CD28 pathway (Fig.3A). Similar downregulation of T cell-dependent cytokine responses was observed after stimulation with another polyclonal T cell activator, Con A. The levels of Th2 cytokines, including IL-4, IL-5, and IL-13, were elevated in the same cultures, indicating that Mst1^−/−^ T cells are committed primarily to the Th2 subtype of helper T cells. Moreover, the production of IL-10, an anti-inflammatory cytokine synthesized by Th2 cells and CD4^+^CD25^+^Foxp3^+^ regulatory T (Treg) cells, was significantly upregulated in Mst1^−/−^ T cells.

To verify these results at a single cell level, we measured cytokine production in CD3/CD28-activated CD4^+^ T cells using flow cytometry analysis of intracellular cytokine staining. As shown in Fig. S2, the percentage of IFN-γ-producing Mst1^−/−^ CD4^+^ T cells was lower than that of WT CD4^+^ T cells, whereas the frequency of IL-13- and IL-10-positive Mst1^−/−^ T cells was elevated compared to WT CD4^+^ T cell cultures. The mean fluorescence intensity (MFI) of IFN-γ-positive cells was also decreased in Mst1^−/−^ CD4^+^ T cells when compared to that of WT cells (data not shown). In agreement with the decreased frequency of IFN-γ-producing T cells, Mst1^−/−^ CD4^+^ T cells cultures contained lower percentages of cells expressing Tbet, a Th1-specific T box transcription factor that controls the expression of IFN-γ and directs Th1 lineage commitment [28]. In contrast, the frequency of cells expressing the canonical Th2 transcription factor GATA3 [29] was increased in Mst1^−/−^ CD4^+^ T cell cultures as compared to that in WT CD4^+^ T cell cultures, suggesting that the reduction in Th1 cytokines and Th2 skewing in CD3/CD28-activated Mst1^−/−^ T cell cultures is due to the reduced number of T cells producing Th1 cytokines and deficient Th1 cytokine synthesis.

Finally, we observed a significant overproduction of IgE in Mst1^−/−^ mice (Fig. 3B), in agreement with the elevated in vitro secretion of Th2 cytokines which control isotype switching to IgE. Serum levels of other Ig isotypes in Mst1^−/−^ mice were similar to controls (data not shown).

We also analyzed the expression of cell activation markers following CD3 stimulation of Mst1^−/−^ T cell cultures. The majority of WT T cells expressed CD69 and CD95 (Fas/APO-1) at 36 hrs post stimulation, in agreement with previously published reports [30], [31] (Fig. 3C). In contrast, the expression of both markers was significantly reduced on Mst1^−/−^ T cells. We did not observe any significant differences in the expression of CD127 (IL-7 receptor α chain), and CD178 (FasL) on CD3-activated splenic T cells from Mst1^−/−^ and WT mice (data not shown).

Next we examined the ability of Mst1^−/−^ T cells to respond to foreign histocompatibility Ags expressed on MHC-mismatched stimulator cells by culturing C57Bl/6-Albino/129SvEv Mst1^−/−^ and WT T cells (H-2b haplotype responders) with irradiated allogeneic Balb/c cells (H-2d haplotype stimulators). The proliferative response of Mst1^−/−^ splenocytes to allogeneic cells was almost undetectable, whereas the proliferation of WT splenic T cells in response to allogeneic stimulation increased in a cell concentration-dependent manner (Fig. 3D).

Taken together, our findings indicate that genetic deletion of Mst1 leads to significant downregulation of Th1 cell-specific cytokine and proliferative responses, including allorecognition. Furthermore, T cell development and cytokine balance in Mst1^−/−^ mice is skewed toward Th2 subtypes producing cytokines with immunosuppressive and anti-inflammatory properties.

### Mst1^−/−^ T cells show delayed cell cycling and undergo apoptosis after activation

**Figure 4:**
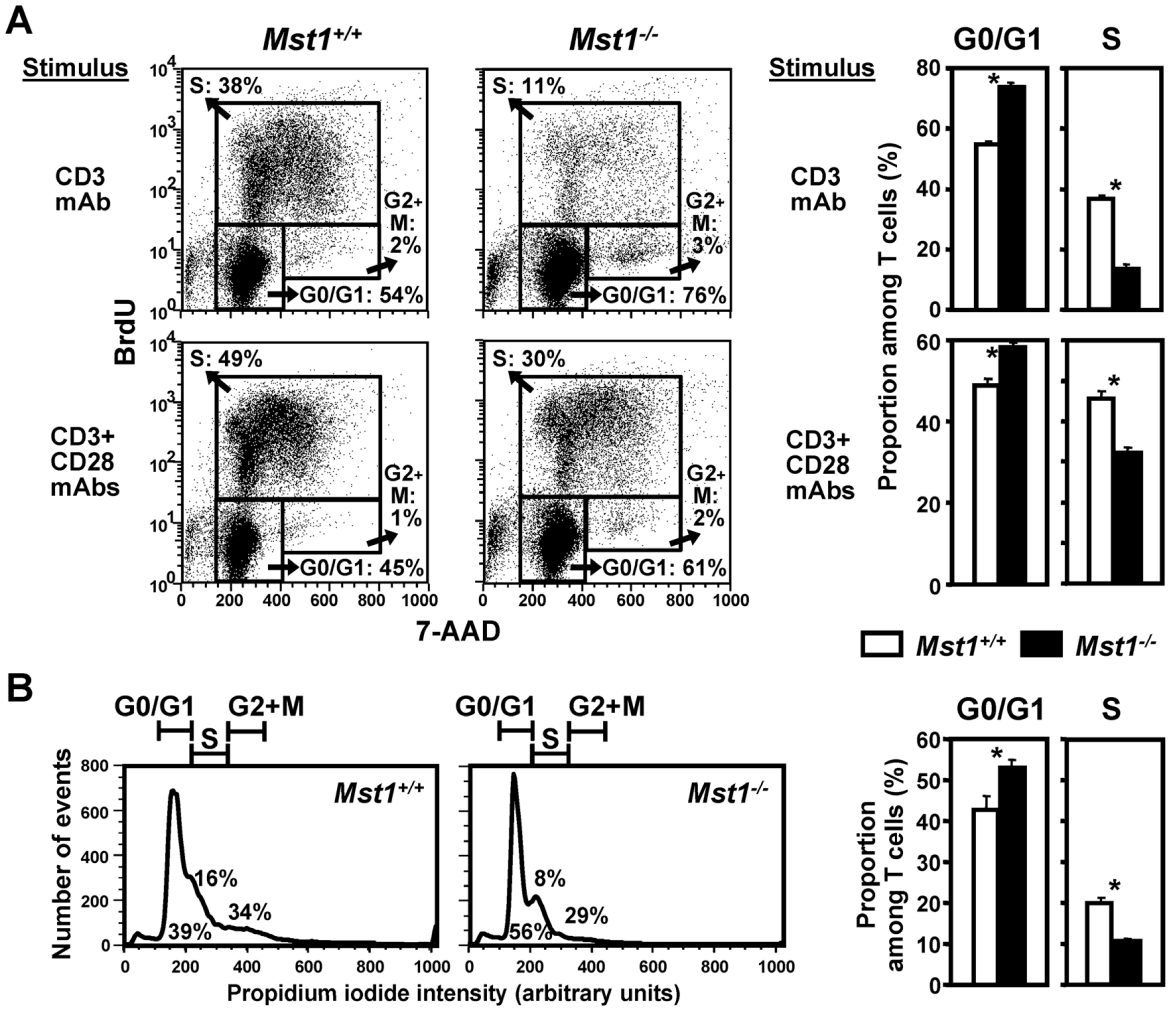
Delayed cell cycle progression of Mst1^−/−^ T cells in vitro. (**A**) Mst1^−/−^ and WT T cells were activated for 36 hrs with the indicated stimuli, pulsed with BrdU, and analyzed by flow cytometry (n = 5 mice per genotype). The representative dot plots and fractional values show cell subsets residing in the indicated phases of cell cycle. (**B**) Nuclear DNA content at different stages of the cell cycle was determined by FACS analysis of splenic T cells stimulated for 48 hrs with plate-bound CD3 mAb (1 µg/ml) and stained with propidium iodide. Histograms are representative of 5 samples per genotype. Values on bar graphs and statistical significance are expressed as in Fig. 2. Similar data were obtained in two additional independent experiments.

**Figure 5:**
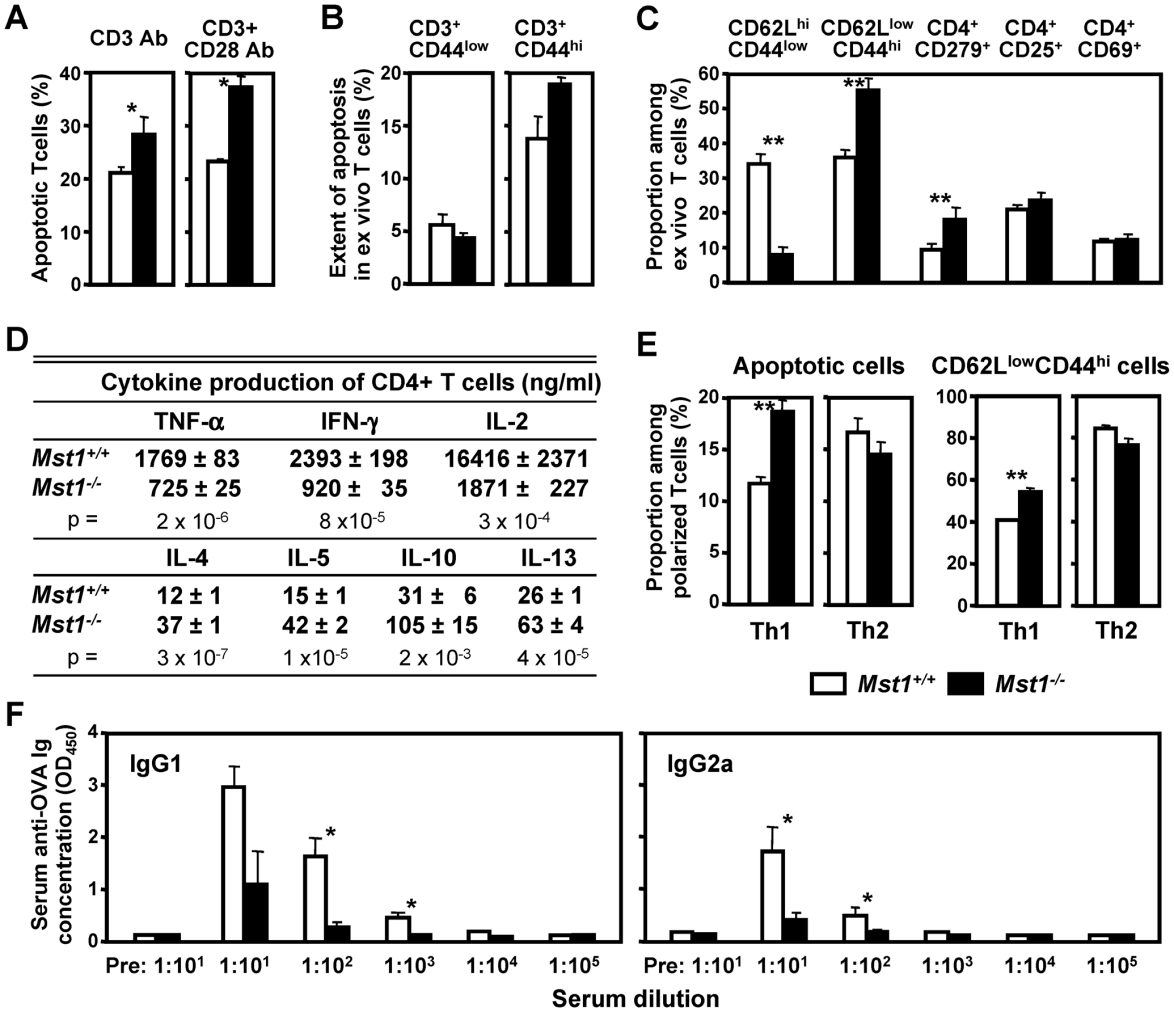
Analysis of T cell apoptosis and functional immune responses in Mst1^−/−^ mice. (**A**) Increased apoptosis of activated Mst1^−/−^ splenic T cells in vitro. Early apoptotic T cells were quantitated by flow cytometry in after stimulation with the indicated mAbs for 48 hrs (n = 5 per genotype). (**B**) Analysis of apoptosis in unstimulated MST^−/−^ T cells. Early apoptotic T cells were quantitated by flow cytometry in CD44^low^ and CD44^high^ subsets of freshly isolated splenocytes (n = 5 per genotype). (**C**) The percentages of naïve (CD62L^high^CD44^low^), effector memory (CD62L^low^CD44^high^), and CD279-, CD25-, and CD69-positive splenic CD4^+^ T cells were examined by flow cytometry (n = 5 per genotype). (**D**) Cytokine production by splenic CD62L^high^CD44^−^ CD4^+^ T cells from WT and Mst1^−/−^ mice (n = 5 per genotype) was examined 48 hrs after stimulation with mAbs to CD3 and CD28 (both at 1 µg/ml). (**E**) Th1 and Th2 polarized cells were generated from naïve splenic CD62L^high^CD44^−^ CD4^+^ T cells after in vitro culture in polarizing conditions for 5 days. The percentages of effector memory (CD62L^low^CD44^high^) CD4^+^ T cells were examined by flow cytometry (n = 5 per genotype). The percentage of sub-G0/G1 apoptotic cells was determined by the BrdU/7-AAD Flow kit and flow cytometry (n = 5 per genotype) following restimulation in vitro with plate-bound CD3 mAb (5 µg/ml) for 48 hrs. (**F**) Mst1^−/−^ deficiency leads to decreased Ag-specific adaptive immune responses in vivo. Mst1^−/−^ and WT mice (n = 6 and 9, respectively) were immunized with OVA in CFA. On day 14, serum samples were analyzed for OVA-specific IgG1 and IgG2a concentrations. Values and statistical significance are expressed as in Fig. 2 and are representative of at least two independent experiments. Pre, preimmune serum.

To investigate whether the deficient lymphocyte proliferation that we observed in Mst1^−/−^ mice were linked to altered cell cycle progression and deficient survival, we evaluated the cell cycle status and apoptosis of activated Mst1^−/−^ T cells. Cell cycle entry, which was induced by CD3 or CD3/CD28 stimulation in WT splenic T cells in a dose-dependent manner, was significantly delayed in Mst1^−/−^ T cells (Fig. 4A and 4B). The percentage of resting G0/G1 Mst1^−/−^ T cells was higher than that of WT T cells, whereas the proportion of Mst1^−/−^ T cells entering the S phase of cell cycle was decreased compared to WT T cells. The 7-AAD-/Annexin V+ T cell population (early apoptotic cells) was significantly increased in Mst1^−/−^ T cell cultures in comparison to WT T cell cultures (Fig. 5A), whereas apoptosis in freshly isolated and unstimulated splenic Mst1^−/−^ T cells was not elevated compared to WT T cells (Fig. 5B), suggesting that the increased cell death in CD3- or CD3/CD28-activated Mst1^−/−^ T cells is not due to an elevated (and preexisting) level of apoptosis of naïve Mst1^−/−^ splenic T cells. In agreement with these findings, we observed a decrease in the expression of bcl-2, an anti-apoptotic protein which enhances the survival of lymphocytes (4), in CD3/CD28-activated Mst1^−/−^ CD4^+^ T cells when compared to that of WT CD4^+^ T cells (Fig. S2).

Further analysis of freshly isolated splenic T cells revealed a significant upregulation of CD279 on the cell surface of naïve Mst1^−/−^ splenic CD4^+^ T cells (Fig. 5C). CD279 (programmed cell death protein 1, PD-1) is a member of the CD28/CTLA-4 family, which negatively regulates T cell-mediated immune responses, including T cell proliferation, cytokine secretion, and primes lymphoid cells to apoptosis [32], [33]. Therefore, naïve Mst1^−/−^ T cells may display altered proliferation and cytokine secretion, and be primed for AICD via upregulation of PD1. Mst1^−/−^ spleens (Fig. 5C), lymph nodes, and peripheral blood (data not shown) also contained higher percentages of CD62L^low^CD44^high^ effector memory T cells, despite their seemingly normal activation status, as evidenced by expression analysis of the T cell activation markers CD25 and CD69. These findings are consistent with the previously published reports [13], [34], [35] and raise the possibility that deficient T cell-mediated responses in Mst1^−/−^ mice are due to higher percentages and elevated AICD of CD62L^low^CD44^high^ T cells. To address this possibility, we examined cell cycle progression and expression of activation markers in purified naïve (CD62L^high^CD44^low^) Mst1^−/−^ and WT CD4^+^ T cells. In agreement with our findings obtained using unfractionated/total T cells, we observed a significant decrease in TNF-α, IFN-γ, and IL-2 secretion and upregulation of Th2 cytokines, including IL-4, IL-5, IL-13, and IL-10 in Mst1^−/−^ naïve CD4^+^ T cell cultures stimulated via CD3/CD28 in nonpolarizing conditions (Fig. 5D), accompanied by a delay in the entry of naïve CD62L^high^CD44^low^ Mst1^−/−^ CD4^+^ T cells into S phase (Fig. S3A). Importantly, a significant decrease in IFN-γ- and Tbet-positive cells was also evident in Mst1^−/−^ naïve (CD62L^high^CD44^−^) CD4^+^ T cell cultures stimulated via CD3/CD28 in nonpolarizing conditions (Fig. S3B), indicating a preferential loss of CD4^+^ T cells producing Th1 cytokines. As expected, the proliferative responses of Mst1^−/−^ naïve splenic CD4^+^ T cells to allogeneic cells were significantly downregulated (Fig. S3C).

These findings indicate that the block in cell cycle progression and deficient T cell activation also occur in naïve Mst1^−/−^ CD4^+^ T cells independently of activation status. However, they do not exclude the possibility of elevated apoptosis in differentiated Th1 cells, which may further contribute to deficient Th1 responses in Mst1^−/−^ mice. To address this scenario directly, we generated WT and Mst1^−/−^ Th1 and Th2 cells by culturing purified naïve (CD62L^high^CD44^low^) CD4^+^ T cells under Th1 and Th2 polarizing conditions. First, we assessed the proportion of IFN-γ^+^IL-4^−^ and IFN-γ^−^IL-4^+^ T cells and the concentration of IFN-γ and IL-4 released upon CD3 restimulation in these cell cultures. Deletion of the Mst1 gene did not affect Th1 and Th2 differentiation in vitro, including the dynamics of cell growth and IFN-γ/IL-4 secretion under Th1 and Th2 polarizing conditions (data not shown). The fact that WT and Mst1^−/−^ cells differentiated equally well under Th1 and Th2 polarizing conditions, rules out the possibility that Mst1^−/−^ T cells are inherently refractory to cytokines that promote Th1 development. Instead, we observed a significant increase in the proportion of CD62L^low^CD44^high^ cells in Mst1^−/−^ Th1 cultures compared to WT cultures. Mst1^−/−^ Th1 cells were also more sensitive to AICD than WT Th1 cells, as evidenced by an increase in the percentage of sub-G0/G1 apoptotic cells following CD3 restimulation in vitro (Fig. 5E). In contrast, the percentages of CD62L^low^CD44^high^ cells and sub-G0/G1 apoptotic cells in Mst1^−/−^ Th2 cultures were similar to those in WT cultures. Thus, the preferential upregulation of CD44 and elevated apoptosis in Th1 cells may contribute to the accumulation of Th2 cells over time and acquisition of the Th2 phenotype in Mst1^−/−^ mice.

### Mst1^−/−^ mice exhibit deficient Ag-specific IgG1 and IgG2a production in vivo

We next studied the effect of Mst1 deletion on Ag-specific T and B cell-dependent adaptive immune responses. To this end, we measured Ag-specific Ig production in Mst1^−/−^ and WT mice following in vivo challenge with OVA in CFA. Mst1^−/−^ mice produced markedly lower amounts of OVA-specific serum IgG1 and IgG2a on day 14 after challenge compared to their WT littermates (Fig. 5F). This observation is consistent with altered in vitro T and B cell function in Mst1^−/−^ mice and provides in vivo evidence that Mst1 plays a critical role in T and B cell-mediated physiological Ag-specific adaptive immune responses.

### Deletion of Mst1 protects against arthritis development in mice

**Figure 6:**
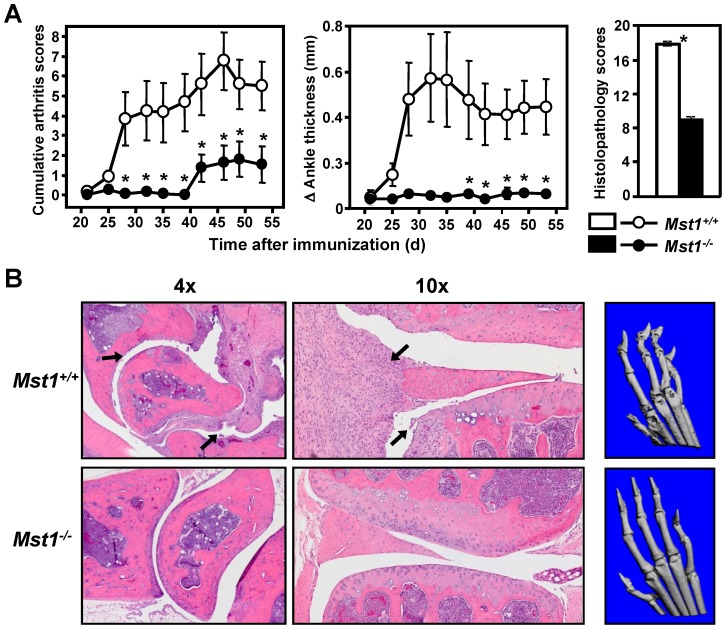
Mst1^−/−^ mice exhibit decreased incidence and severity of CIA. (**A**) Mice of indicated genotype (n = 11–13) were immunized with CII in CFA and observed for clinical signs of arthritis at the time points depicted on the X axis. Histological scores of synovial inflammation and cartilage/bone erosion were analyzed on day 45 after immunization. Data are expressed as mean (± SEM); * (p<0.05) indicates significant differences in comparison to WT littermates (Mann–Whitney U test). Similar data were obtained in two additional independent experiments. (**B**) Representative pictures of histological and radiographic signs of arthritis in the same mice as in (A), obtained on day 45 after immunization with CII (left and middle panels). Arrows of the H&E-stained sections of paw joints point to severe synovial inflammation and cartilage erosion in the Mst1^−/−^ animals. Representative µCT images of subchondral bone changes characteristic of arthritis were taken on day 45 after immunization (right panels).

To study the role of Mst1 in autoimmune mechanisms involved in rheumatoid arthritis, we immunized Mst1^−/−^ mice with CII emulsified in CFA, using a modification of the standard immunization procedure successfully adapted by Campbell et al. to induce CIA in mice derived from the C57BL/6 (H-2^b^) genetic background, including C57BL/6×129/Sv hybrids [36]. Consistent with the above findings of decreased T cell- and B cell-mediated immune responses in the absence of Mst1, the Mst1^−/−^ mice displayed a markedly decreased incidence and severity of arthritis when compared with their WT littermates (Fig. 6A). Moreover, Mst1 deficiency significantly reduced histological signs of arthritis, including synovial inflammation and cartilage/bone destruction (Fig. 6A and 6B). Further differences in the severity of arthritis between Mst1^−/−^ and WT mice were revealed by three-dimensional X-ray tomography (µCT; Fig. 6B). WT CIA mice showed severe joint abnormalities, including i. bone erosions and decalcification localized in or adjacent to the involved paw joints, ii. joint space widening, suggesting the presence of inflammatory exudate, and iii. signs of ligamentous or capsular laxity leading to joint subluxations. In contrast, µCT analysis of the affected joints revealed only minor arthritis-associated degenerative changes in Mst1^−/−^ mice.

These findings demonstrate that Mst1 plays an important role in autoimmunity and regulates susceptibility to CIA. Downregulation of proinflammatory cytokines may contribute to the diminished inflammatory immune responses in Mst1^−/−^ mice immunized with CII.

### Deletion of Mst1 reduces the severity of EAE in homozygous animals

**Figure 7:**
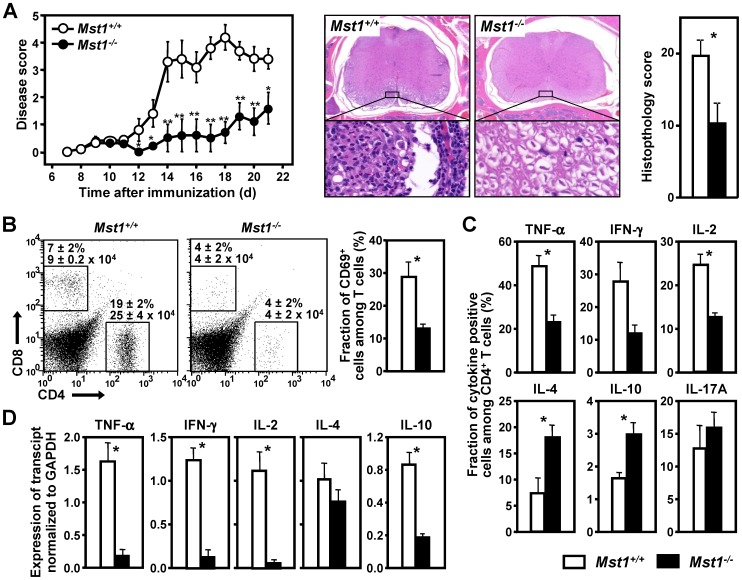
Mst1^−/−^ mice are resistant to EAE. (**A**) EAE was induced in mice of indicated genotype (n = 10) by immunization with MOGp35–55 in CFA. The mean EAE score ± SEM observed in each group is plotted against time after immunization (left panel). Representative H&E stained sections of the cervical spinal cord of WT and Mst1^−/−^ mice obtained on day 21 after immunization (middle panels). Higher (×40) magnification shows areas of prominent mononuclear multifocal inflammation, vacuolization and gliosis in the white matter of WT mice, which are nearly absent in Mst1^−/−^ mice. Aggregate histopathology scores were obtained on day 21 after immunization (right panel). Similar data were obtained in two additional independent experiments. * (p<0.05) and ** (p<0.01) indicate significant differences in comparison to WT animals (Mann–Whitney U test). (**B**) Infiltrating mononuclear cells were isolated from the spinal cord of mice with the indicated genotype (n = 5/group) on day 15 after immunization with MOGp35–55, and analyzed by flow cytometry for markers of T cell differentiation and activation. Numbers above the rectangular gates represent the percentages and absolute numbers (x10^4^/spinal cord) of infiltrating CD4^+^ and CD8+ T cells for each genotype. (**C**) Frequency of IL-2-, TNF-α-, IFN-γ-, IL-4-, IL-10, and IL-17A-producing CD4^+^ T cells infiltrating the spinal cords of indicated mice was measured by flow cytometry analysis of intracellular cytokine staining. (**D**) qPCR analysis of IL-2, TNF-α, IFN-γ, IL-4, and IL-10 mRNA in the spinal cords from mice of indicated genotype (n = 6–10) was performed on day 11 after immunization with MOGp35–55. Values are expressed relative to the expression of GAPDH. *Values and statistical significance are expressed as in Fig. 2 and represent at least two independent experiments.*

EAE is an animal model of multiple sclerosis, which can be induced in mice by active immunization with the encephalitogenic fragments of myelin proteins [37], [38], [39]. We immunized Mst1^−/−^ and ^+/+^ littermates with myelin oligodendrocyte glycoprotein peptide 35–55 (MOGp35–55) prepared in CFA (Fig. 7A). The onset of disease was significantly delayed in Mst1^−/−^ animals (p = 0.0098, as determined by the Kaplan-Meier log rank test). In the post-immunization phase of EAE (days 7–22), we observed a 4-fold decrease in the mean cumulative disease scores in Mst1^−/−^ mice compared with their WT littermates (8.1±3.6 vs. 31.8±2.5 in Mst1^−/−^ and WT mice, respectively; p = 0.0017 by the Mann–Whitney U test).

Detailed histological analysis of brain and spinal cord sections revealed that the severity of inflammatory and degenerative lesions was significantly lower in Mst1^−/−^ mice compared to WT controls (Fig. 7A). Overall, Mst1^−/−^ mice were significantly less likely to develop multifocal lymphocytic meningitis, which was a prominent and uniform finding in the spinal cord of WT mice immunized with MOGp35–55. In addition, cytometric analysis of mononuclear cells infiltrating the spinal cord of immunized mice showed a marked decrease in the absolute number of CD4^+^ T cells and the percentage of activated CD69^+^ T cells in the Mst1^−/−^ mice at the peak of disease (Fig. 7B). The later results demonstrate a critical role of Mst1 in the accumulation of activated T cells in target CNS tissues. Importantly, the frequency of IL-10- and IL-4-producing CD4^+^ T cells in the spinal cords of Mst1^−/−^ EAE mice was significantly higher than that of WT EAE controls, whereas the percentages of IL-2-, TNF-α-, and IFN-γ-producing CD4^+^ T cells infiltrating the spinal cords of Mst1^−/−^ mice was significantly reduced compared with their WT littermates (Fig. 7C). Interestingly, the spinal cords of Mst1^−/−^ and WT EAE mice contained similar percentages of IL-17A-positive CD4^+^ T cells, suggesting a limited role for Mst-1 in driving IL-17-medited immune responses in this disease model. Despite the increase in the frequency of IL-4- and IL-10-producing CD4^+^ T cells in the CNS of Mst1^−/−^ mice, the absolute numbers of both Th1- and Th2-secreting CD4^+^ T cells were lower in Mst1^−/−^ spinal cords (data not shown), in agreement with the significant decrease in the absolute number of total CD4^+^ T cells infiltrating the CNS of Mst1^−/−^ EAE mice (Fig. 7B). Consistent with the lower degree of CD4^+^ T cell infiltration, qPCR analysis revealed markedly decreased levels of IL-2, TNF-α, and IFN-γ mRNA in the spinal cords of Mst1^−/−^ EAE mice (Fig. 7D). In contrast, comparable levels of IL-4 mRNA were observed in spinal cord samples from Mst1^−/−^ and WT EAE mice, supporting a Th2-skewed cytokine response in the CNS tissues of Mst1^−/−^ mice immunized with MOGp35–55.

**Figure 8:**
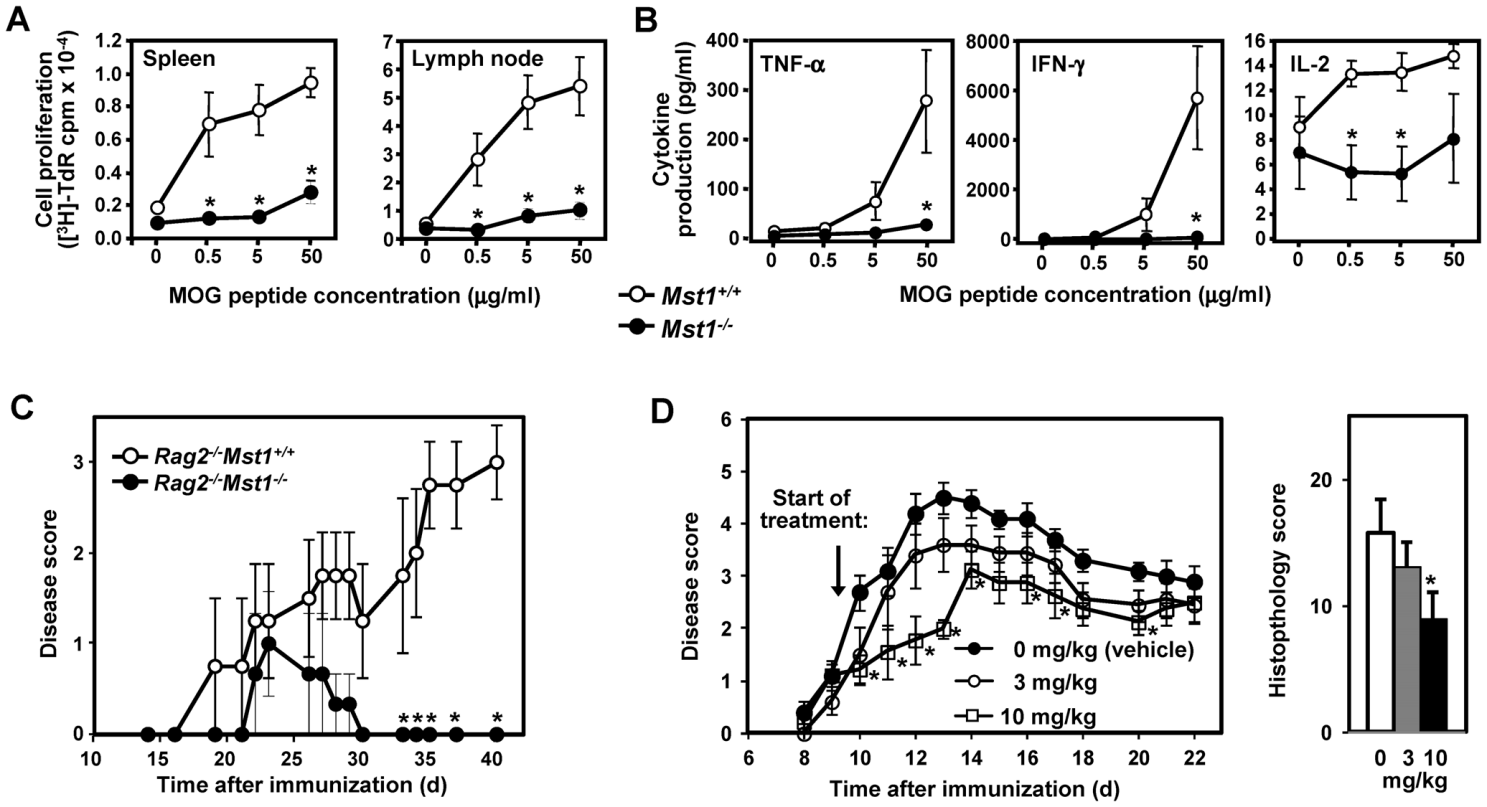
Mst1^−/−^ mice exhibit blunted MOG-specific proliferative and effector cytokine recall responses in vivo. Splenocytes and lymph node cells isolated from MOGp35–55-immunized Mst1^−/−^ and WT mice (day 11 after immunization; n = 5 per genotype) were incubated with MOGp35–55 at the indicated concentrations. (**A**) Cell proliferation was assessed by [^3^H]-thymidine incorporation after 72 hrs of stimulation. (**B**) Cytokine production was examined 48 hrs after stimulation with MOGp35–55. The results shown are expressed as in Fig. 2 and are representative of two independent experiments. (**C**) Clinical EAE scores in Rag2^−/−^ mice reconstituted with WT (n = 5) or Mst1^−/−^ (n = 5) CD4^+^ T cells. Purified WT or Mst1^−/−^ CD4^+^ T cells were transferred into Rag2^−/−^ mice, which were subsequently immunized with MOG35–55 in CFA. Data are presented as in Figure 7A and are representative of two experiments. (**D**) Mice treated with an Mst1 inhibitor exhibit decreased severity of EAE. EAE was induced in C57Bl/6-Albino/129SvEv mice (n = 10 per treatment group) by immunization with MOGp35–55 in CFA and treated twice daily with the indicated doses of the Mst1 inhibitor LP-945706 administered by oral gavage in citrate buffed (pH 4.5, 0.1 M) 1% Tween 80 vehicle, starting one day after the onset of disease in 50% of the animals. Mean EAE scores ± SEM (plotted against time after immunization) and aggregate histopathology scores (day 22 after immunization) were analyzed for statistical differences between treatment groups using the Kruskal-Wallis non-parametric analysis of variance (ANOVA) with Dunnett's multiple-comparison post hoc analysis (* p<0.05 compared to vehicle control). Data are representative of two experiments.

To determine whether the priming of myelin epitope–specific peripheral CD4^+^ T cells is deficient in Mst1^−/−^ mice following immunization with encephalitogenic peptides (i.e., during the induction/sensitization phase of EAE), we measured cell proliferation and cytokine production of lymph node and splenic cells isolated from MOGp35–55-immunized Mst1^−/−^ and WT mice and restimulated ex vivo with MOGp35–55. As shown in Fig. 8A and 8B, the T cell-dependent recall response to MOGp35–55, including cell proliferation and secretion of IL-2 and effector cytokines such as TNF-α and IFN-γ, was markedly reduced in Mst1^−/−^ mice. These results suggest that the delayed onset and reduced severity of EAE in Mst1^−/−^ mice is mediated by deficient T cell sensitization against encephalitogenic Ags and altered differentiation of T cells into effector cells during the induction phase of EAE.

### Rag2^−/−^ mice reconstituted with Mst1^−/−^ CD4^+^ T cells are resistant to EAE

The EAE-resistant phenotype of Mst1^−/−^ mice may potentially be attributed to the lymphopenia and/or alterations in other immune cell types involved in EAE progression. To examine the contribution of a T cell-intrinsic defect to resistance to EAE in Mst1^−/−^ mice, we used an adoptive transfer system where EAE was induced in Rag2^−/−^ recipient mice that received equal numbers of WT or Mst1^−/−^ CD4^+^ T cells. As shown in Fig. 8C and Fig. S4A, all the mice transferred with WT CD4^+^ T cells developed clinical signs of EAE after immunization with MOG35–55 peptide, whereas only one out of five Mst1^−/−^ CD4^+^ T cell recipients had transient symptoms of EAE. Importantly, the frequencies of total CD4^+^ and activated CD25^+^ CD4^+^ T cells were markedly decreased in the spinal cord of the Rag2^−/−^ recipients that received Mst1^−/−^ CD4^+^ T cells as compared with those transferred with WT CD4^+^ cells (Fig. S4B). Taken together, these results indicate that Mst1^−/−^ CD4^+^ T cells display an intrinsic defect in their ability to respond to encephalitogenic antigens and that deletion of Mst1 in the CD4^+^ T cell compartment is sufficient to alleviate CNS inflammation during EAE. Of note, sublethally irradiated WT animals reconstituted with syngeneic Mst1^−/−^ bone marrow cells exhibit substantially lower disease severity and incidence compared to animals reconstituted with WT bone marrow (K. Salojin, unpublished observation), providing further evidence for an important role of the lymphoid compartment in the resistance of Mst1^−/−^ mice to autoimmunity.

### Severity of EAE is alleviated in mice treated with an Mst1 inhibitor

The resistance of the Mst1 knockout to a number of autoimmune models has prompted the discovery of novel compounds that are potent inhibitors of Mst1 [40]. The primary biochemical and cell-based assays used for high-throughput screening and lead optimization are described in the Supplementary Materials and Methods (File S1). As an example, LP-945706, a small molecule inhibitor identified from lead optimization, inhibited purified Mst1 at 1 mM ATP with an IC50 of 2.3 nM and intracellular Mst1 with an IC50 of 7 nM (Fig. S5A and S5C). The compound also inhibited IFN-γ, TNF-α, and IL-2 production by T cells in a dose-dependent manner with IC50 values of 0.31, 0.22, and 0.13 µM, respectively (Fig. S5C). To address whether LP-945706 is selective for MST1 over other kinases, LP-945706 was screened against 456 kinases by DiscoveRx (Fremont, CA) in the absence of ATP (data not shown). From this screen, assays were developed in-house against kinases with highest affinity for LP-945706. As shown in Figure S5B, at physiological ATP (1 mM) LP-945706 exhibited between 23 and >2000 fold selectivity for Mst1 over the other kinases tested. More specifically, the selectivity panel showed that LP-945706 inhibited Mst1 with about 23-fold greater potency than MST2, a related MAP4K kinase (HGK), and Abl kinase; besides these three kinases, LP-945706 had greater than 100-fold selectivity over all other kinases.

Next we evaluated the compound in a mouse EAE model for multiple sclerosis. Dose selection was based on the cellular IC50 value for Mst1, the IC50 values in the in vitro cytokine production assay, and drug concentration in the plasma (corrected for plasma protein binding). Fig. 8D shows the effect of different oral doses of LP-945706 when administered therapeutically to mice with EAE induced by immunization with MOGp35–55 peptide. The inhibitor significantly decreased the mean EAE clinical scores in the 10 mg/kg twice daily dose group, as compared with animals treated with the vehicle control. We observed a ∼40–50% decrease in the mean disease scores in EAE mice treated with the Mst1 inhibitor dosed orally at the 10 mg/kg dose. Plasma exposures (Cp) of compound at the measured Tmax (1 hr post dose) are shown in Fig. S5D. At 1 hour post dose, the Cp of LP-945706 in the 10 mg/kg group was 286 nM that correlated to a free fraction concentration of 22 nM. The free fraction of LP-945706 that gives ∼40–50% efficacy was therefore in between the IC50 observed in the Mst1 cell-based assay and the aforementioned cytokine production assays. At the same time, free drug concentrations in the 3 and 10 mg/kg groups stayed at least several fold below IC50 values for the kinases listed in Fig. S5B.

## Discussion

The relatively restricted expression pattern of the Mst1 gene in lymphoid/myeloid cells [12], [13] suggests that the primary physiological role of Mst1 is associated with development and function of the immune system. Our analysis of genetic deletion of Mst1 establishes a central and nonredundant role for Mst1 in controlling T cell- and B cell-mediated adaptive immune responses, allorecognition, and autoimmunity.

It is recognized that Mst1 may exhibit both proapoptotic and antiapoptotic functions in vitro depending on the cell type and the context in which cell activation occurs. Specifically, Mst1 may deliver proapoptotic signals by phosphorylating H2B, participating in caspase cascade, and activating the MKK4/JNK signaling pathway [4], [6], [7], [8], [9], [10], [11]. On the other hand, in vivo findings indicate that Mst1 plays an important role in lymphocyte survival, regulation of cellular oxidative stress, and maintenance of naive T cell homeostasis in the periphery [13], [18].

Two recent reports shed light on the role of Mst1 in human lymphocyte function by describing a primary immunodeficiency phenotype associated with homozygous mutations in the Mst1 gene [34], [35]. Both reports provided more evidence of antiapoptotic functions of Mst1 in human lymphocytes by demonstrating progressive loss of T and B cells in patients lacking Mst1, due to excessive apoptosis in lymphocytes, including anti-Fas-mediated apoptosis, and altered expression of the IL7 receptor and bcl-2 [34], [35]. Our findings in Mst1^−/−^ mice showed that T and B cell proliferation, T cell-mediated cytokine production, and expression of T cell activation markers were greatly reduced in the absence of Mst1. The impaired functional responses of Mst1^−/−^ T cells were observed in association with severely impaired IL-2 production, altered Th1/Th2 balance, and elevated apoptosis of Mst1^−/−^ Th1 cells, which coincided with (1) the accumulation of CD62L^low^CD44^high^ effector memory T cells in peripheral lymphoid tissues of Mst1^−/−^ mice, and (2) a partial block in cell cycle progression in naïve Mst1^−/−^CD4^+^ T cells. The increased cell death in activated Mst1^−/−^ T cells in vitro was not due to the higher initial percentage of apoptotic cells in naïve splenic T cells, and the block in cell cycle progression and deficient T cell activation also occurred in naïve Mst1^−/−^CD4^+^ T cells. Our results suggest that the upregulation of CD44 and CD279 in Mst1^−/−^CD4^+^ T cells may directly downregulate T cell responses and lead to elevated AICD. CD279 is known to negatively regulate T cell-mediated immune responses, including T cell proliferation, IL-2 and IFN-γ secretion [32], [33], whereas upregulation of CD44 on peripheral Mst1^−/−^ T cells may prime them to Fas-mediated apoptosis, in keeping with the role of CD44 as a physiological trigger of Fas upregulation and AICD [41], [42], [43]. In agreement with this scenario, splenocytes from CD44 KO mice demonstrated resistance to TCR-mediated apoptosis and exhibited increased delayed-type hypersensitivity responses [42], [44]. In addition, the elevated apoptosis of Mst1^−/−^ T cells in response to CD3/CD28 stimulation may be due to altered survival mechanisms, including deficient bcl-2 expression.

Rapid and efficient IL-2 release by CD4^+^ T cells is crucial to maintaining T cell survival, proliferation, synthesis of other T cell cytokines, and differentiation of T cells into effector T cells (reviewed in [45]). IL-2 acts through its receptor (IL-2R) to activate positive feedback signaling mechanisms that further amplify T cell responses to IL-2. Therefore, the failure of naïve Mst1^−/−^ CD4^+^ T cells to upregulate IL-2 production and expression of the α chain of the IL-2R (CD25) during the primary response to CD3/CD28 stimulation (Fig. 5D and S3B) may be one of the earliest events that result in deficient T cell responses in Mst1^−/−^ mice.

Given the significant reduction in cytokine production, particularly IL-2, we hypothesized that the proliferation defects in T cells can be rescued with IL-2 supplementation. Indeed, exogenous IL-2 significantly enhanced Mst1^−/−^ T cell proliferation, resulting in a partial rescue of IFN-γ synthesis by Mst1^−/−^ T cells; however, IL-2 failed to completely rescue the proliferative defect and elevated apoptosis in CD3/CD28-stimulated Mst1^−/−^ T cells (K. Salojin, unpublished observation). These results suggest that the deficient IL-2 production by CD4^+^ T cells is only one potential mechanism of suppressed immune responses in Mst1^−/−^ mice.

Impaired Th1 cytokine secretion in Mst1^−/−^ mice was observed in association with a Th2 skewing of cytokine production leading to elevated production of Th2 cytokines in vitro, and overproduction of Th2-dependent IgE in vivo, suggesting that Mst1 controls Th1/Th2 balance. Our analysis of cytokine production at a single cell level indicate that the reduction in Th1 cytokines and Th2 skewing in CD3/CD28-activated Mst1^−/−^ T cell cultures may be due to deficient Th1 cytokine synthesis and/or a preferential loss of CD4^+^ T cells producing Th1 cytokines, accompanied by accumulation of Th2 cytokine-producing CD4^+^ T cells. The increased sensitivity of Th1 cells to apoptosis that we observed in Mst1^−/−^ mice may have contributed to a shift from a Th1 to a Th2 cytokine profile and acquisition of the Th2-like phenotype. Importantly, the preferential AICD of Th1 effector cells in mixed Th1/Th2 cultures was described as a mechanism of differential regulation of Th1/Th2 development leading to selective Th2 survival [46], [47]. The suppression of Th1 cytokine secretion in Mst1^−/−^ mice is also accompanied by a significant increase in the production of IL-10, an anti-inflammatory cytokine synthesized by Th2 cells and CD4^+^CD25^+^Foxp3^+^ natural Tregs. The elevated production of IL-10 is likely mediated by the Th2 skewing of cytokine production, as there was no defect in the development of CD4^+^CD25^+^Foxp3^+^ Tregs in Mst1^−/−^ mice (K. Salojin, unpublished observation). Interestingly, IL-10 mRNA levels are also significantly elevated in activated CD4^+^ T cells from Jak3^−/−^ mice [48], underscoring the significant similarities in the phenotype observed after genetic deletion of Mst1 and Jak3.

Accumulation of CD62L^low^CD44^high^ T cells in peripheral lymphoid organs has been observed in various immunodeficiencies, including Jak3- and common cytokine receptor γ chain (γ_c_)-deficient mice [48], [49], [50]. Jak3^−/−^ T cells are functionally unresponsive and this signaling defect is associated with deficient proliferation and IL-2 secretion. In addition, both resting and activated Jak3^−/−^ and γ_c_^−^ peripheral T cells are much more prone to apoptosis due to impaired survival mechanisms, including deficient bcl-2 expression [48]. The increased apoptosis of Jak3^−/−^ T cells in response to TCR ligation contributes to the deficient cytokine production observed in Jak3^−/−^ mice, as Jak3^−/−^ T cells tend to die rapidly upon activation [48]. In view of the lymphodepletion observed in Mst1^−/−^ and Jak3^−/−^ mice, the accumulation of CD62L^low^CD44^high^ T cells in the peripheral lymphoid organs may be driven by a mechanism of proliferative expansion compensating for the increased apoptosis and impaired IL-2 production in T cells. A similar functional T cell phenotype was described in mice homozygous for a single tyrosine (Y136F) mutation in LAT (linker for activation of T cells) [51]. LATY136F mutant CD4^+^ T cells proliferate poorly in vitro in response to CD3/CD28 stimulation despite their CD62L^low^CD44^high^ activation status, and exhibit a Th2 skewing.

The Jak3^−/−^ and γ_c_^−/−^ immunological phenotypes are primarily mediated by deficient negative selection of self-reactive T cells in the thymus and the periphery [48], [49], [50]. We and others observed the accumulation of mature CD4 and CD8 single-positive T cells in the thymus of Mst1^−/−^mice due to altered egress of mature thymocytes [16], [17], and K. Salojin, unpublished observation). However, we observed no major differences in Vβ usage in the splenic and lymph node CD4^+^ subsets from Mst1^−/−^ mice (K. Salojin, unpublished observation). These results suggest that the T cell phenotype of Mst1^−/−^ mice is not mediated by altered peripheral TCR repertoire and is not a consequence of expansion of autoreactive clones.

Despite the apparent skewing toward a Th2-like cytokine profile, we observed a significant decrease in the Ag-specific immune responses of Mst1^−/−^ mice, including deficient IgG1 and IgG2a production in vivo, and failure to respond to allogeneic cells in the mixed lymphocyte reaction. The impaired secretion of IL-2 and altered T and B cell function in Mst1^−/−^ mice are likely responsible for these functional defects. These functional defects coupled with the acquisition of a Th2-skewed phenotype with immunosuppressive and anti-inflammatory properties are consistent with the resistance of Mst1^−/−^ mice to disease induction in a number of Th1-mediated autoimmune and inflammatory disease models. Mst1^−/−^ mice develop less severe EAE with significantly lower disease scores and much less evident multifocal lymphocytic meningitis and degenerative lesions in the CNS. Various proinflammatory and Th1-dependent cytokines, such as IL-12, IFN-γ and TNF-α play a role in the pathogenesis of multiple sclerosis by promoting myelin sheath injury. By contrast, IL-10, IL-13, and IL-27 dampen the detrimental immune responses to the CNS self Ags [52], [53], [54]. Our observations underscore the importance of Mst1 signaling in T cells in controlling differentiation of peripheral T cells into effector cells during the sensitization phase of EAE. Despite of this partial peripheral sensitization block, some Mst1^−/−^ CD4^+^ T cells retain their capacity to differentiate into effector cells capable of infiltrating into the spinal cord; however, the effector phase of EAE in Mst1^−/−^ mice is associated with a Th2 skewing of cytokine production leading to severely impaired IL-2 and Th1 cytokine secretion in the infiltrated CNS. Deficient activation of infiltrating T cells, as evidenced by a decrease in the number of activated CD69^+^ T cells in the CNS of Mst1^−/−^ mice with EAE, may further contribute to reduced local expansion of Ag-driven CD4^+^ T cells in the target tissues.

To confirm that resistance of Mst1^−/−^ mice to EAE is a direct result of a functional defect intrinsic to the T cell compartment, we examined the induction of EAE in Rag2^−/−^ mice reconstituted with either WT or Mst1^−/−^ CD4^+^ T cells. Our results demonstrated that CD4^+^ T cell intrinsic defects are responsible for attenuated autoimmunity in Mst1^−/−^ mice and provide further evidence for an essential and non-redundant role of Mst1 expressed in the CD4^+^ T cell compartment in EAE progression.

In addition to its role in lymphocyte survival, Mst1 is involved in various aspects of LFA-1-mediated adhesion and trafficking, including lymphocyte homing to target organs and thymocyte emigration [14], [15], [16], [17]. In agreement with this model, CD4^+^ T cells lacking LFA-1 show deficient Ag-dependent activation and cytokine production in vivo [55], whereas blocking high affinity LFA-1 inhibits activation of naïve T cells [56]. Attenuated CD4^+^ T cell infiltration of the CNS tissues of Mst1^−/−^ mice immunized with MOGp35–55 suggests that LFA-1-mediated activation and migration of primed T lymphocytes to the CNS parenchyma may be another mechanism that can be impaired in Mst1^−/−^ mice, and it is currently under investigation in our laboratory.

Proinflammatory and Th1-dependent cytokines produced by infiltrating cells play a important role in the pathogenesis of rheumatoid arthritis by increasing osteoclast activity in the joints (reviewed in [57], [58], [59]. Abnormal apoptosis and increased proliferation of inflammatory cells, including T lymphocytes, have also been implicated in autoimmune arthritis [60], [61]. Our results show that deletion of Mst1 protects against the development of systemic autoimmune arthritis and blocks lymphocyte-mediated inflammation in the synovium leading to the destruction of cartilage and underlying bone. Together, these in vivo observations underscore the essential role of Mst1 in T cell-mediated adaptive in vivo responses to self Ags in autoimmune diseases.

The Mst1 kinase emerges as a critical regulator of lymphocyte function and autoimmunity. Targeting Mst1 and Mst1-mediated signaling pathways that control Th1-dependent cytokine release, IL-2 secretion, and lymphocyte proliferation represents a promising alternative to the current approaches to treat autoimmunity, organ rejection and graft-versus-host disease. The findings described in this manuscript have prompted us to search for novel compounds that are potent inhibitors of Mst1, exhibit desirable pharmacokinetic properties, and are effective in animal disease models. The compound described in this report shows greater than 23-fold selectivity for MST1 over all kinases in the kinome and inhibits IFN-γ, TNF-α, and IL-2 production by T cells in a dose-dependent manner. In addition, we observed a significant increase in AICD in WT T cells treated with Mst1 inhibitors, coinciding with downregulation of Th1-specific cytokines (K. Salojin, unpublished observation). However, the impact of the compound-mediated pharmacological inhibition of Mst1 on Th1 and Th2 cell differentiation in vivo and in vitro is unknown and is currently under investigation in our laboratory. Moreover, based on the analysis of the primary potency and kinase selectivity of this compound we cannot completely rule out the possibility that some of the in vitro effects of the inhibitor are mediated by off-target inhibition of other related kinases.

Consistent with the compound-mediated in vitro inhibition of cytokine production, we observed a decrease in EAE clinical and histological scores in the treatment group with drug concentrations at or above the cellular IC50 value for Mst1. At the same time, drug concentrations in the 3 and 10 mg/kg groups stayed several fold below IC50 values for related MAP4K kinases, including Mst2, HGK, and at least 10-fold below that of unrelated kinase families (Src protein tyrosine kinases, Janus kinases, cyclin-dependent kinases, etc.). These results suggest that the mitigation of clinical EAE scores in this study was primarily driven by inhibition of Mst1. However, based on the data presented in this report, we cannot completely rule out that some of the in vivo efficacy in this model was due to at least partial off-target inhibition of one or more of the other kinases. The precise mechanism(s) by which these inhibitors alleviates EAE severity and the role of Mst1-specific inhibition in disease response will be revealed by further studies and PK/PD modeling.

## Supporting Information

Figure S1 **Loss of Mst1 expression and unaltered levels of Mst2 in Mst1^−/−^ splenocytes.** Splenocytes and kidney cells isolated form mice (WT and Mst1^−/−^) or Lewis rats were lysed in Triton X-100 lysis buffer containing protease inhibitors, and analyzed by western blot for expression of Mst1 and Mst2. β-actin was used as a control for equal protein loading. Results are representative of two independent experiments.
(TIF)

Figure S2 **Frequency of IFN-γ-, IL-13-, IL-10, Tbet-, GATA3-, and bcl-2-expressing WT and Mst1^−/−^ CD4^+^ T cells.** Purified CD4^+^ T cells pooled from 4–6 WT and Mst1^−/−^ animals were left unstimulated (0 hr) or stimulated with mAbs to CD3 and CD28 (both at 1 µg/ml) for the indicated time periods, and analyzed by FACS for expression of various intracellular markers depicted on the figure. Results are representative of two independent experiments.
(TIF)

Figure S3 **In vitro analysis of cell cycle and functional immune responses mediated by naïve (CD62L^high^CD44^−^) Mst^−/−^ CD4^+^ T cells.** (**A**) Flow cytometric analysis of lymph node (LN) and splenic CD62L^high^CD44^−^ CD4^+^ T cells of indicated genotype (n = 5/genotype) stimulated for 60 hrs with mAbs to CD3 and CD28 (both at 1 µg/ml) and pulsed with BrdU. Dot plots show cell subsets residing in the indicated phases of cell cycle. Values on bar graphs and statistical significance are expressed as in Fig. 2. (**B**) Naïve CD62L^high^CD44^−^ CD4^+^ T cells pooled from 4-7 WT and Mst1^−/−^ animals were left unstimulated (0 hr) or stimulated with mAbs to CD3 and CD28 (both at 1 µg/ml) for the indicated time periods, and analyzed by FACS for expression of various intracellular markers depicted on the figure. Activation of CD62L^high^CD44^−^ CD4^+^ T cells was assayed by surface staining for CD25. (**C**) Proliferation of splenic CD62L^high^CD44^−^ CD4^+^ responder T cells (H2b) after stimulation with the indicated numbers of MHC-mismatched (H-2d) irradiated stimulator cells. Results are expressed as the mean ± SEM cpm values of triplicate cultures and are representative of at least two independent experiments.
(TIF)

Figure S4 **Flow cytometric analysis of spleens and spinal cords from Rag2−/− mice reconstituted with WT or Mst1−/− CD4+ T cells.** (**A**) FACS analysis of reconstitution efficiency in Rag2^−/−^ mice that received WT or Mst1^−/−^ CD4^+^ T cells. Splenocytes of either naïve (non-immunized) WT and Rag2^−/−^ controls or non-immunized Rag2^−/−^ mice reconstituted with WT or Mst1^−/−^ CD4^+^ T cells were analyzed for expression of the indicated T cell-specific markers on day 10 after CD4^+^ T cell transfer (n = 2 per group). The percentages (top dot plot panels) and absolute numbers (x10^6^/spleen; bottom panel) of TCRβ^+^ CD4^+^ T cells for each experimental group were quantitated by FACS. (**B**) Rag2^−/−^ mice reconstituted with WT or Mst1^−/−^ CD4^+^ T cells were immunized MOGp35–55 in CFA as described in Fig. 8C. Infiltrating mononuclear cells isolated from the spinal cord of the animals were assayed by flow cytometry (n = 5/group; the cells were pooled for analysis). Numbers inside the dot plots represent the percentages and absolute numbers (x 10^4^/spinal cord) of infiltrating CD4^+^ and CD25^+^ CD4^+^ T cells. Results are representative of two independent experiments.
(TIF)

Figure S5 **Potency and selectivity of LP-945706.** (**A**) Representative dose response curves for LP-945706 in the primary biochemical (open circles) and cell-based (closed circles) assays for Mst1. The IC50 of purified Mst1 by LP-945706 was measured using a Z′-Lyte assay that monitors phosphorylation of a FRET-peptide substrate in the presence of physiological ATP (1 mM). The cell-based assay is based on autophosphorylation on intracellular Mst1 and the IC50 was determined as described in the Supporting Materials and Methods (File S1). (**B**) Kinase selectivity data for LP-945706. A Z′-Lyte assay (File S1) was used for measuring IC50 values of all kinases shown except BIKE and ALK6; for the latter two kinases, a P81 assay was developed that monitors incorporation of [33P]-ATP into a protein substrate. All IC50 measurements shown were determined for purified kinases in the presence of 1 mM ATP. Values shown are averages from at least two separate experiments. (**C**) Mean IC50 values for LP-945706 in the Mst1 autophosphorylation cell-based assay (File S1) and T cell-mediated cytokine production assay [40]. For the MST1 cell-based assay, the IC50 value is an average of ten separate experiments, whereas the cytokine IC50 values are an average of two separate experiments. (**D**) Plasma concentration of LP-945706 at 1 hr post-dose (Tmax) in the mouse EAE model was measured by liquid chromatography–tandem mass spectrometry as described in the Supporting Materials and Methods (File S1). Values are expressed as mean ± SD (n = 10 per treatment group) and are representative of two experiments.
(TIF)

File S1 **Supporting materials and methods.**
(DOCX)
