# Supplementary figures and images for: Genetic Deletion of Mst1 Alters T Cell Function and Protects against Autoimmunity
Source: PLoS One. 2014 May 22;9(5):e98151. doi: 10.1371/journal.pone.0098151 (PMC4031148; doi:10.1371/journal.pone.0098151)

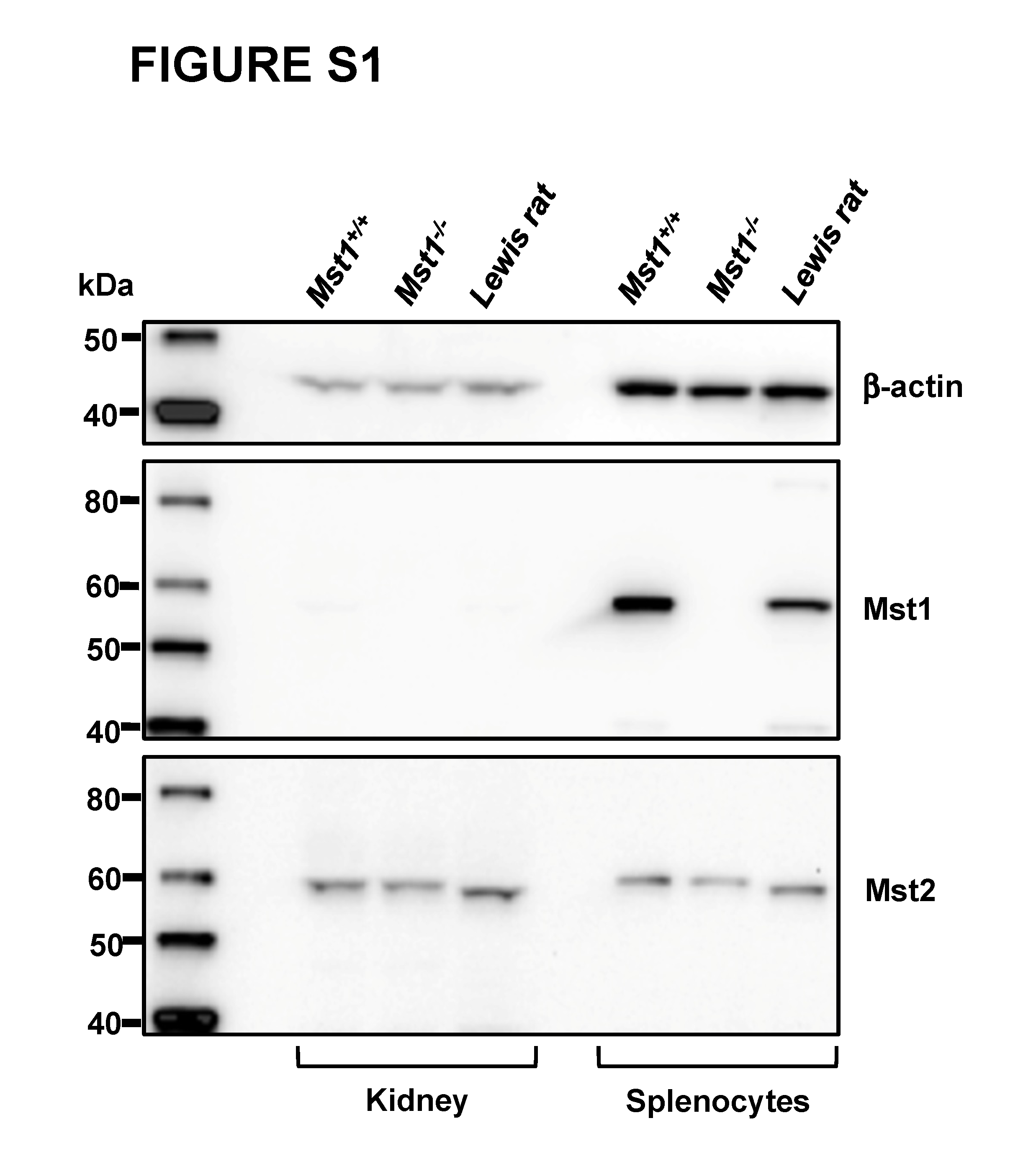

Supplement: Figure S1 — Loss of Mst1 expression and unaltered levels of Mst2 in Mst1−/− splenocytes. Splenocytes and kidney cells isolated form mice (WT and Mst1−/−) or Lewis rats were lysed in Triton X-100 lysis buffer containing protease inhibitors, and analyzed by western blot for expression of Mst1 and Mst2. β-actin was used as a control for equal protein loading. Results are representative of two independent experiments. (TIF) [file pone.0098151.s001.tif]

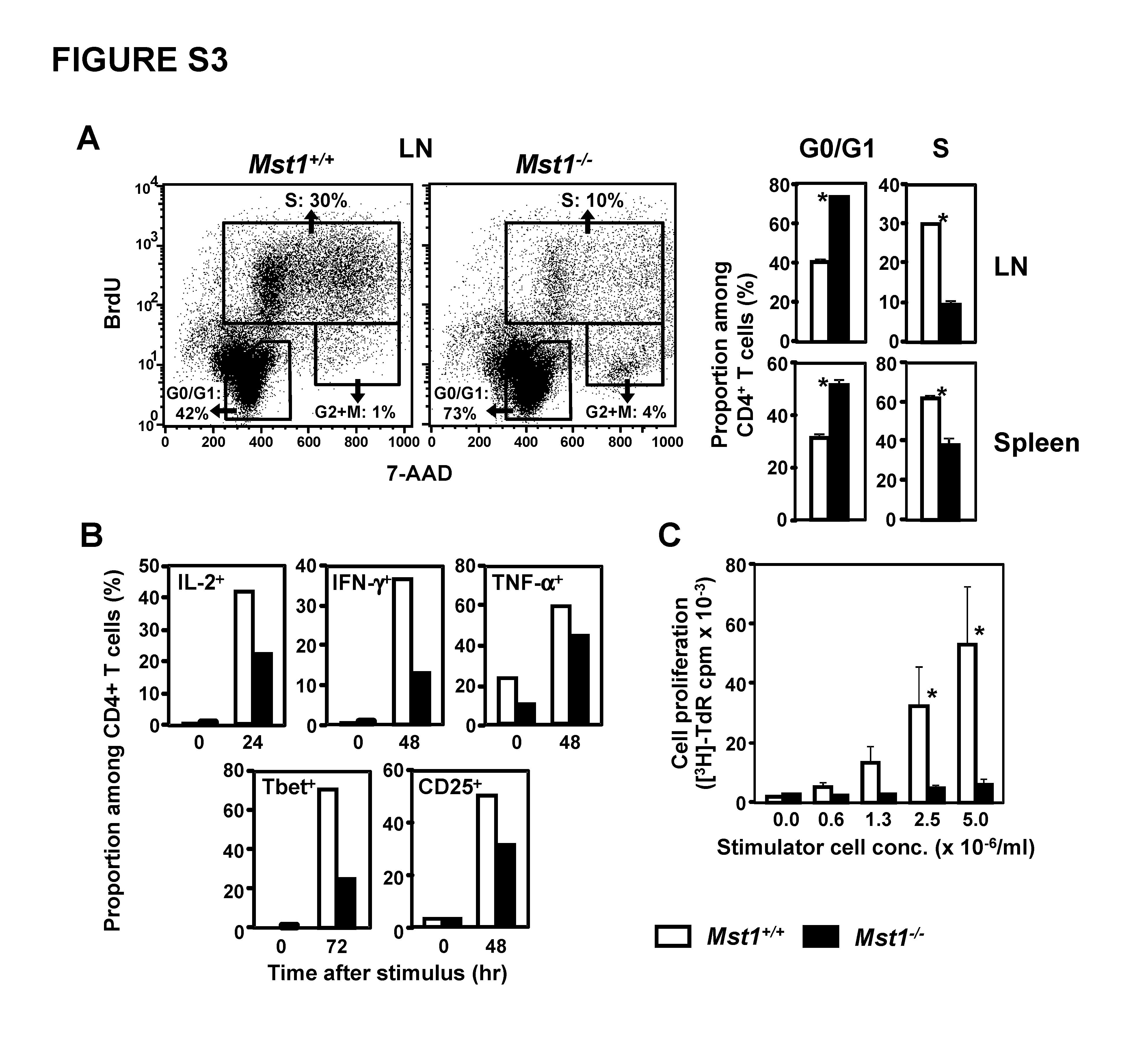

Supplement: Figure S3 — In vitro analysis of cell cycle and functional immune responses mediated by naïve (CD62LhighCD44−) Mst−/− CD4+ T cells. (A) Flow cytometric analysis of lymph node (LN) and splenic CD62LhighCD44− CD4+ T cells of indicated genotype (n = 5/genotype) stimulated for 60 hrs with mAbs to CD3 and CD28 (both at 1 µg/ml) and pulsed with BrdU. Dot plots show cell subsets residing in the indicated phases of cell cycle. Values on bar graphs and statistical significance are expressed as in Fig. 2. (B) Naïve CD62LhighCD44− CD4+ T cells pooled from 4-7 WT and Mst1−/− animals were left unstimulated (0 hr) or stimulated with mAbs to CD3 and CD28 (both at 1 µg/ml) for the indicated time periods, and analyzed by FACS for expression of various intracellular markers depicted on the figure. Activation of CD62LhighCD44− CD4+ T cells was assayed by surface staining for CD25. (C) Proliferation of splenic CD62LhighCD44− CD4+ responder T cells (H2b) after stimulation with the indicated numbers of MHC-mismatched (H-2d) irradiated stimulator cells. Results are expressed as the mean ± SEM cpm values of triplicate cultures and are representative of at least two independent experiments. (TIF) [file pone.0098151.s003.tif]

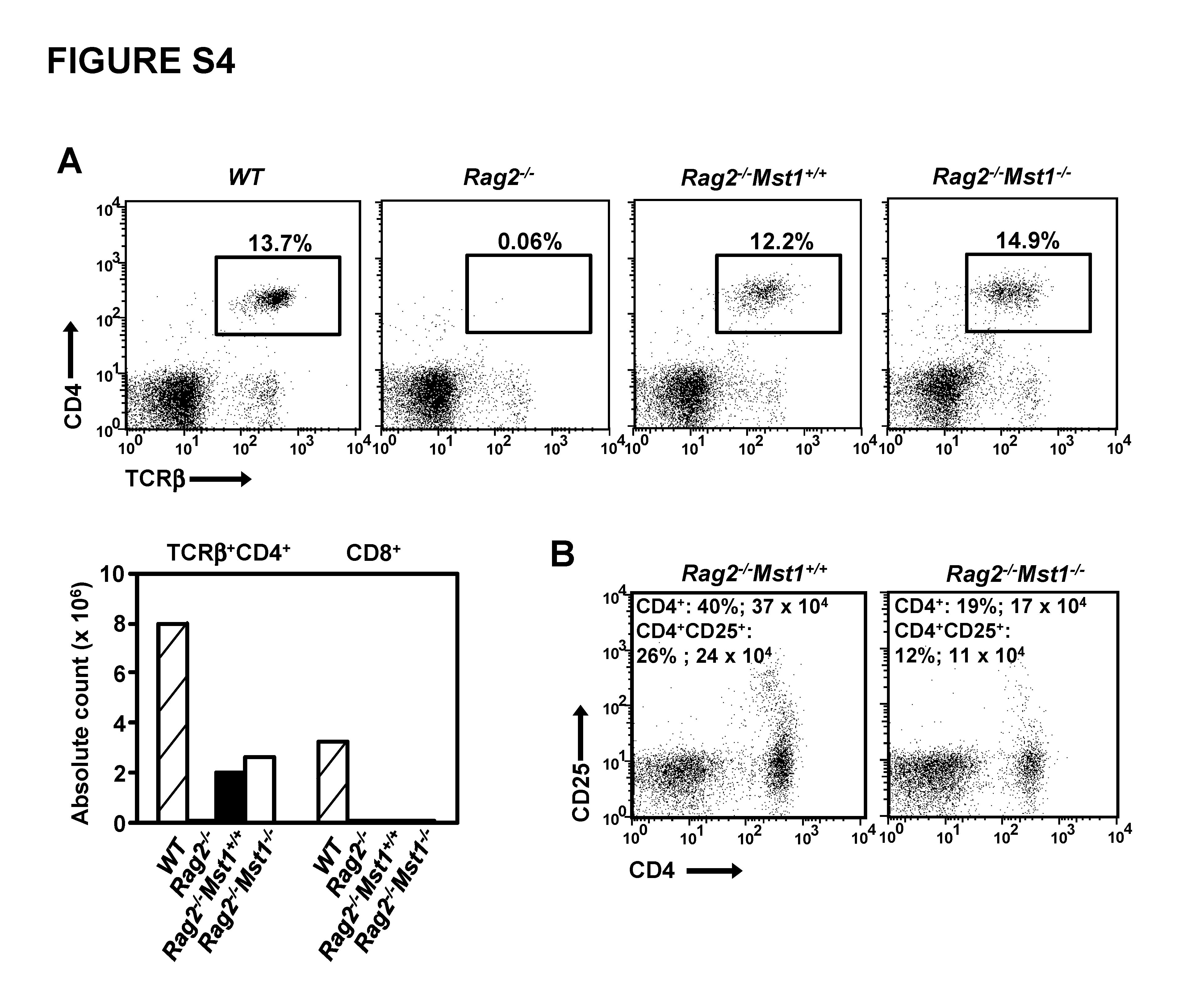

Supplement: Figure S4 — Flow cytometric analysis of spleens and spinal cords from Rag2−/− mice reconstituted with WT or Mst1−/− CD4+ T cells. (A) FACS analysis of reconstitution efficiency in Rag2−/− mice that received WT or Mst1−/− CD4+ T cells. Splenocytes of either naïve (non-immunized) WT and Rag2−/− controls or non-immunized Rag2−/− mice reconstituted with WT or Mst1−/− CD4+ T cells were analyzed for expression of the indicated T cell-specific markers on day 10 after CD4+ T cell transfer (n = 2 per group). The percentages (top dot plot panels) and absolute numbers (x106/spleen; bottom panel) of TCRβ+ CD4+ T cells for each experimental group were quantitated by FACS. (B) Rag2−/− mice reconstituted with WT or Mst1−/− CD4+ T cells were immunized MOGp35–55 in CFA as described in Fig. 8C. Infiltrating mononuclear cells isolated from the spinal cord of the animals were assayed by flow cytometry (n = 5/group; the cells were pooled for analysis). Numbers inside the dot plots represent the percentages and absolute numbers (x 104/spinal cord) of infiltrating CD4+ and CD25+ CD4+ T cells. Results are representative of two independent experiments. (TIF) [file pone.0098151.s004.tif]
