# Supplementary material for: Genetic Deletion of Mst1 Alters T Cell Function and Protects against Autoimmunity
Source: PLoS One. 2014 May 22;9(5):e98151. doi: 10.1371/journal.pone.0098151 (PMC4031148; doi:10.1371/journal.pone.0098151)
Supplement: File S1 — Supporting materials and methods. (DOCX) [file pone.0098151.s006.docx]

SUPPORTING MATERIALS AND METHODS

*Western blotting*

Cells were lysed in ice-cold 20 mM Tris, pH 7.5, 150 mM NaCl, 1mM EDTA, 1 mM EGTA lysis buffer containing 1% Triton X-100, 5% glycerol (all obtained from Sigma-Aldrich), and supplemented with a mixture of protease and phosphatase inhibitors (Halt protease and phosphatase inhibitor cocktail; Thermo Fisher Scientific). Lysates were incubated on ice for 15 min, clarified of detergent-insoluble material by centrifugation (10 min, 14,000 rpm), and normalized for protein concentration levels. Equal amounts of total protein were separated by SDS-PAGE, transferred to a PVDF membrane, blotted with indicated antibodies, and visualized by the Pierce ECL substrate (Thermo Fisher Scientific). Signal intensities of specific bands were visualized and quantified using a VersaDoc imaging system (Bio-Rad, Hercules, CA). The antibodies used were: anti-β-actin (Sigma-Aldrich; catalog # A2066), anti-Mst1 (Cell Signaling Technology, Bevery, MA; catalog # 3682), or anti-Mst2 (Novus Biologicals, Littleton, CO; catalog # NB110-57229).

*In vitro measurement of Mst1 inhibition*

A Z’-Lyte assay based on a fluorescence resonance energy transfer (FRET) readout was developed for measuring Mst1-dependent phosphorylation of a FRET-peptide substrate*. Assays were performed in low-volume 384-well black proxiplates (PEBlk-Proxi-6008269; Perkin Elmer, Akron, OH). Eight compounds were first diluted into Low Density Volume plates (Labycte, Sunnyvale, CA) using a Multiprobe II Plus HTEX reagent dispenser (model # AMP8E01; Perkin Elmer, Akron, OH) at a starting concentration of 1 mM compound in 100% DMSO. Using a ECHO 550 acoustic dispenser (Labcyte), 75 nl compound was pinged into 384-well proxiplates. The starting concentration of compound in each assay plate was 7.5 µM, followed by 3-fold dilutions, to give quadruplicate ten-point concentration curves. Five µl of a 2× solution of Mst1 enzyme in kinase buffer (Life Technologies) was added to wells containing compound or DMSO controls, followed by a 10 min preincubation step. Reactions were initiated by adding 5 μl of a mixture of ATP and Z’-Lyte Ser/Thr 7 peptide (Life Technologies) and proceeded at RT for 1 hr. The final concentration of key reagents in the kinase reactions were 1 μM substrate, 1 nM enzyme, and 1 mM ATP. At the end of the kinase reaction, 10 µl of developing solution (Life Technologies; catalog # PR4876B) was added to each well and incubated at RT for 1 hr. All wells were read on an Envision 2101 Multilabel fluorescence plate reader (Perkin Elmer) at 400 nm excitation and 460 nm/530 nm emission. Plus and minus enzyme controls were used to calculate percent inhibition and IC50 curves were generated using Microsoft Excel.

* Rodems SM, Hamman BD, Lin C, Zhao J, Shah S, Heidary D, Makings L, Stack JH, and Pollok BA (2002) A FRET-Based In Vitro Assay for both Tyrosine- and Serine/Threonine-Specific Kinases and Phosphatases. Assay Drug Dev. Technol. 1: 9-20.

*Kinase selectivity assays*

All kinases were purchased from Life Technologies and specific catalog numbers are shown below. Z’-Lyte assays were used for each of the kinases except ALK6 and BIKE. For the Z’-Lyte assays, the protocol was identical to that described for Mst1 except slight variations in enzyme concentration and the Z’-Lyte peptide substrate (Life Technologies) as follows: 1 nM Mst2, Ser/Thr 7 Peptide; 0.3 nM HGK (PV3687), Ser/Thr Peptide 7; 5 nM TYRO3 (PV3828), Tyr 2 peptide (PV3191); 0.5 nM SRC (P3044), Tyr 2 peptide; 3 nM JAK2 (PV4210), Tyr 6 peptide (PV4122); 0.5 nM JAK3 (PV3855), Tyr 6 peptide; 0.5 nM FGR (P3041), Tyr 2 peptide; 2 nM CDK2 (PV3267), Ser/Thr 13 peptide (PV3793); 0.5 nM CDK1 (PV3292), Ser/Thr 13 peptide; 2 nM AXL (PV3971); 0.1 nM ABL (P3049), Tyr 2 peptide. For ALK6 and BIKE, assays were developed using a standard radiometric assay involving phosphocellulose 384-well plates (Millipore, County Cork, Ireland). For ALK6, 2 nM kinase (SignalChem, BC, Canada; catalog # B05-11G-10) was used along with 40 µM TGFBR1 peptide (SignalChem) as the substrate. BIKE kinase was expressed and purified in-house (manuscript in preparation) and the assay included 2 nM enzyme and 0.5 µM Mu2 protein (expressed and purified in-house) as the substrate.

*Cell-based assay*

A cell-based assay that monitors autophosphorylation of intracellular Mst1 was developed. In this assay, HEK293F cells (Life Technologies) were transfected with a plasmid coding for full-length human Mst1 (accession # Q13043). The cells were grown in cell media (DMEM, 10% FBS, 1× GPS (L-Glutamine-Penicillin-Streptomycin; all from Gibco Life Technologies) to 80-90% confluency in 24-well culture plates. On day one, ~2 x 10^7^ cells were trypsinized and resuspended in 20 ml cell media minus GPS. One ml of this cell suspension was transferred to a Vi-cell sample cup. Cells were counted and then diluted to 3 x 10^5^/ml in DMEM supplemented with 10% FBS. Each well of a 24-well plate received 500 μl of this cell suspension for a final concentration of 1.5 x 10^5^ cells per well. Cells were then incubated overnight at 37°C and 5% CO_2_. On day two, each well was transfected with 0.5 μg DNA and 1.5 μl Lipofectamine 2000 (Life Technologies), according to the manufacturer’s instructions. The DNA mix was comprised of 5 ng Mst1 T183E (a mutant form of Mst1 in which Thr183 was replaced with a Glu to enhance detection of a C-terminal phosphorylation site), 445 ng pcDNA3.1 (Life Technologies), and 50 µl OPTI-MEM (Life Technologies). DNA transfection mixture was added to cells in each well of a 24-well plate, followed by incubation overnight at 37°C and 5% CO_2_. On day 3, compounds were serially diluted with 100% DMSO. Next, media/transfection mix was aspirated, followed by addition of 300 μl of each compound DMSO stock diluted 1:1000 in DMEM containing 0.5% FBS. Plates were then incubated at 37°C and 5% CO_2_ for 4 hr, and okadaic acid (0.75 μM) was added to the cultures for 2 more hrs to enhance signal of phosphorylated Mst1 (pMst1). Cells were pelleted by centrifugation at 1000 x *g* for 5 minutes and frozen at -80°C. Cell lysates were prepared for western blot analysis as described above and mixed with 4× LDS sample buffer containing 50 mM DTT (Life Technologies). Samples were denatured at 70°C for 10 min on a PCR machine (PTC-200; MJ Research, Waltham, MA), separated by SDS-PAGE, transferred to a PVDF membrane, blotted with indicated antibodies, and visualized with ECL reagent (GE Healthcare, Pittsburgh, PA) on a Versadoc Imaging System (Model 5000; Bio-Rad). Volume analysis performed on each band to obtain density values. Band intensities of background subtracted pMst1 were normalized for total expression of Mst1 and a “no compound” control was used as the 0% inhibition control. Ratios of pMst1/Mst1 were used to generate IC50s in Microsoft Excel. The antibodies used were: rabbit anti-Mst1 (Millipore; catalog # 07-061); rabbit anti-phospho-Mst1 (Cell Signaling Technology; catalog # 3681); α-rabbit-HRP (Bio-Rad; catalog # 170-6515).

*Pharmacokinetics and liquid chromatography–tandem mass spectrometry (LC-MS/MS)*

Plasma samples were analyzed by LC-MS/MS using a Surveyor HPLC with a TSQ triple quadrupole mass spectrometer coupled with an electrospray ionization (ESI) source (Thermo Fisher Scientific). XCalibur 2.0 SR2 software (Thermo Fisher Scientific) was used for instrument control and data calculation. Calculations were made by normalizing the area counts of analyte to the area counts of the internal standard (verapamil). Samples were quantitated against a standard curve over the concentration range 1.3-10,000 ng/ml with the limit of quantitation of 1.3 ng/ml. Pharmacokinetic parameters were calculated by noncompartmental analysis using Phoenix WinNonlin software (Pharsight, version 6.3).
